# The control of malaria vectors in rice fields: a systematic review and meta-analysis

**DOI:** 10.1038/s41598-022-24055-2

**Published:** 2022-11-16

**Authors:** Kallista Chan, Christian Bottomley, Kazuki Saito, Jo Lines, Lucy S. Tusting

**Affiliations:** 1 Department of Disease Control, London School of Hygiene & Tropical Medicine, Keppel Street, London, UK; 2 Centre On Climate Change and Planetary Health, London School of Hygiene & Tropical Medicine, London, UK; 3 Department of Infectious Disease Epidemiology, London School of Hygiene & Tropical Medicine, London, UK; 4 Africa Rice Center, Bouake, Côte d’Ivoire

## Abstract

The relatively stable aquatic conditions of irrigated lowland and rainfed rice, which is grown across 145 million hectares in more than 100 countries, are capable of generating large numbers of mosquito vectors of malaria, which causes more than 400,000 deaths per year worldwide. Many methods can control these vectors, but a systematic review has not previously been conducted. This study assesses whether larviciding, fish or intermittent irrigation can significantly reduce malaria vectors in rice fields whilst increasing rice yield. After a literature search for studies reporting the effect of larval control and rice cultivation practices on malaria vector densities in rice fields, 33 studies were eligible for meta-analysis. Larviciding was effective at reducing rice-field malaria vectors. Pooled analysis of five controlled time-series (CTS) studies with chemical insecticides showed an overall combined reduction of larval densities of 77% compared to no larviciding. Eight CTSs with biological larvicides showed a pooled reduction of 60% compared to no larviciding. Cultivating rice and fish together provided good control too: a pooled analysis of three CTSs showed an overall 82% reduction in anopheline larvae compared to no fish. Pooled analysis of four studies suggested that intermittent irrigation (using various timings and frequencies of drainage) is effective at reducing the abundance of late-stage anopheline larvae (pooled reduction = – 35%), but not overall immature abundance, compared to continuous flooding. We conclude that many interventions such as larvicides, fish and intermittent irrigation can provide riceland malaria vector control, but the critical obstacle to wider use is farmer acceptability. Future research should be led by the agricultural sector, with inputs from entomologists, to investigate malaria control co-benefits within high-yielding rice cultivation practices.

Rice is one of the major food grains of the world, acting as a staple food crop for about half of the world’s population. Demand for rice is ever-increasing, especially in Africa, with continental production having increased 117% in the last 20 years^1^. In order to keep up with such demand and achieve self-sufficiency, there has been enormous investment of resources towards boosting rice production, including the expansion of rice-harvested areas^2,3^.

Unfortunately, in addition to providing food security and improved farmer livelihoods, irrigated and rainfed lowland rice production systems also generate a large number of mosquitoes. Depending on the region where rice is grown, different sets of mosquito species can be found inhabiting the water, and in some parts of the world, rice fields are a major source of the most important malaria vector species of that region^4^. Examples include central China, sub-Saharan Africa (SSA), and parts of central Asia, Indonesia and Peru, where rice-cultivating areas can produce very high densities of competent malaria vectors, with adult female mosquitoes being up to tenfold more abundant than in neighbouring areas without rice cultivation^4–7^. Thus, rice-growing areas can have high inherent malaria transmission capacity, posing a major public health problem. In many previously malarious countries such as Portugal, Spain, Turkmenistan and China, rice areas were identified as the last hotspots of transmission, and targeted control of mosquito breeding in the rice fields was often required to achieve malaria elimination and to prevent resurgence^8–11^. This rice-malaria relationship is especially important in SSA because African vectors are extraordinarily efficient at transmitting malaria. More than 80% of the world’s 627,000 deaths due to malaria occur in African children under five years of age^12^. There is recent evidence that in Africa, there is a significant association between rice and intensified malaria transmission, and this association has grown stronger over time^15^.

For these reasons, interventions to suppress vector breeding in rice fields have been studied since the 1930s. Malariologists have investigated many methods of larval source management (LSM) in rice fields (e.g. the use of chemical and biological larvicides) and, sometimes in collaboration with agronomists, different agricultural techniques (e.g. irrigation method, plant height and pesticide use). Reviews written over 30 years ago concluded that these interventions have mixed effects on malaria vector densities and that despite numerous studies, there are still major gaps in our understanding of what works, when and where^4,16,17^. In most cases, these reviews presented experimental trials in rice fields as individual case studies without any pooled effect measures. They also rarely included the effect of these interventions on rice production and water consumption as well as the technology readiness of the intervention (i.e. the farmers’ propensity to adopt and incorporate a technology within their rice cultivation practices), all of which are priorities to agronomists when considering methods of rice cultivation.

To measure the success of a rice-based intervention, malariologists are most interested in the epidemiological impact of the vectors coming from rice fields over a cropping season. However, epidemiological outcomes such as malaria prevalence and incidence of neighbouring rice communities are difficult to collect since treatments in rice fields would need to have been implemented on a large-scale (perhaps as a randomised controlled trial) that spanned across entire irrigation schemes. Moreover, due to mosquito flight range and migration, their effects can be difficult to measure; it is not easy to distinguish an epidemiological event caused by a malaria vector originating from rice fields as opposed to a vector from other breeding sites. For the same reasons, comparing adult vector abundance across different communities is not an adequate measure. An alternative measure is the abundance of mosquitoes newly emerged from rice fields, but they are also difficult to collect: it remains challenging to attract large densities of mosquitoes into a trap from such extensive areas^18^. Whilst pupal densities would also have been a more adequate measure, their numbers are small, which can increase sampling error. Thus, malariologists usually resorted to larval density (which often included pupae) as a proxy for adult vector abundance and malaria prevalence. Additionally, the effect of an intervention on larval abundance was often measured in terms of an immediate effect, which did not reveal how persistent the intervention can be over an entire rice-growing season. All things considered, larval density was the main measure of intervention effectiveness in this review.

As an update and supplement to the previous narrative reviews, we conducted a systematic review and meta-analysis to assess whether, by and large, riceland LSM and rice cultivation practices can reduce malaria vector abundance, whilst increasing rice yield and reducing water use.

## Results

### Search results and study characteristics

**Figure 1:**
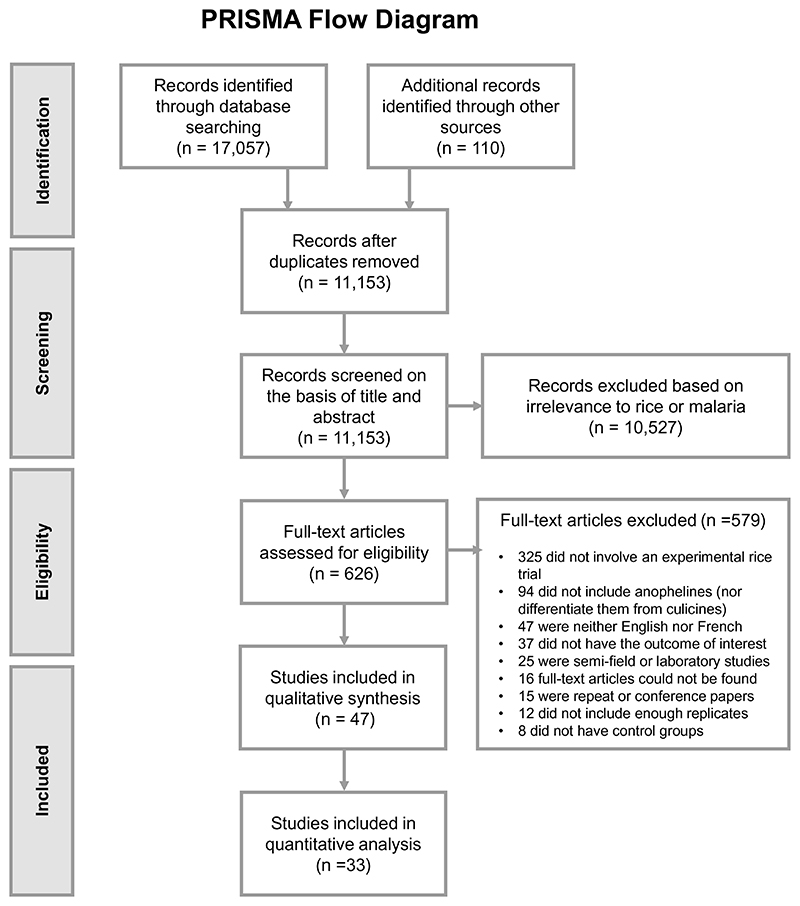
Study selection process.

The literature search yielded 11,153 studies after removing duplicates (Fig. 1). From these, 47 publications were eligible for inclusion. All 47 were included for qualitative analysis, while 33 were included for quantitative analysis, of which 26 were controlled time series (CTS) and 7 were controlled interrupted time series (CITS) studies. Data in CTS studies are collected at the same multiple time points in control and intervention groups only after treatment application whereas data in CITS studies are collected both before and after treatment application(s) (Supplementary Fig. 1)^22^. In total, since studies often tested multiple interventions, there were 84 comparisons. Table 1 summarises all eligible studies (some repeated as they had multiple comparisons) by interventions, publication period and geographical region. Most studies were conducted between 1981 and 2000 (66%) and in America (n = 21, all in USA), followed by Africa (n = 13) and South Asia (n = 12, all in India).

### Risk of bias

High risk of bias was found across numerous domains of the EPOC risk of bias for CTS studies, particularly for allocation concealment (where technicians and investigators could foresee intervention assignment) and blinding (Supplementary Table 1). Amongst the seven CITS studies, there was a high risk of bias for both allocation sequence generation (where non-random methods were used) and allocation concealment. Another common design weakness is a general lack of information on baseline features in both CTS and CITS studies.

There were insufficient studies (n < 10) to construct funnel plots and test for asymmetry for most meta-analyses except for studies that looked at larvicides or water management techniques. Regression tests for funnel plot asymmetry found no evidence for publication bias for the meta-analyses on chemical insecticides (Supplementary Fig. 2A) or water management techniques (Supplementary Fig. 2B). However, there was evidence of publication bias for the meta-analyses of CTS studies on bacterial larvicides (p = 0.02, Supplementary Fig. 2C).

### Larviciding

Compared to no monomolecular surface films (MSF), MSFs for riceland vector control were not associated with reduced anopheline immature densities in one CITS study but were associated with a 57% reduction in anopheline immatures in two CTS studies (95% confidence interval [CI] 69.4, 40.3, p < 0.0001, Table 2). Taking larval stages into consideration, MSFs were associated with a 50% reduction in early instar anophelines and a 55% reduction in late instars (Supplementary Table 2).

**Figure 2:**
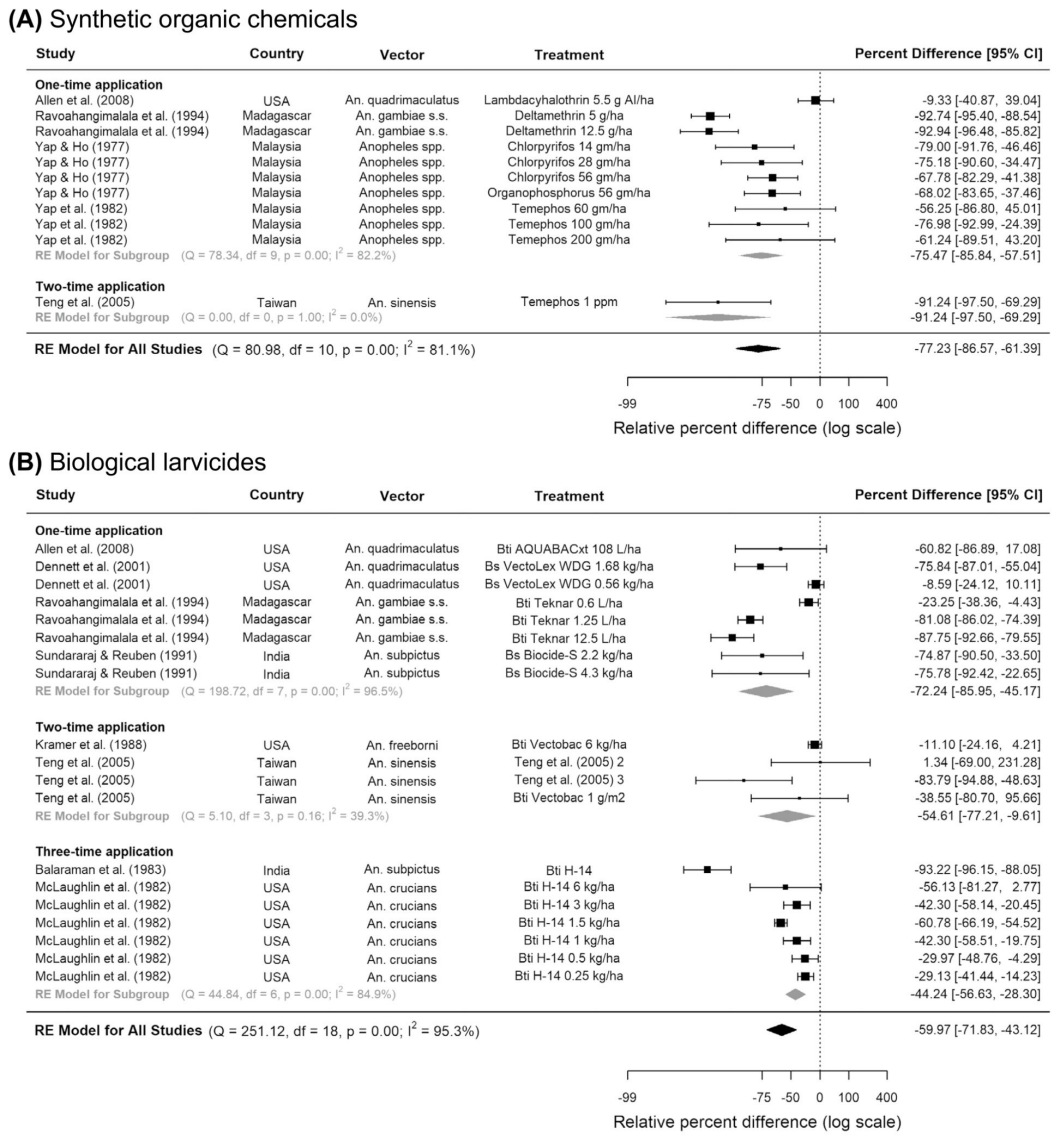
Pooled estimate of the effect of (**A**) synthetic organic chemicals and (**B**) biological larvicides on *Anopheles* larval densities in rice fields. Five controlled time series studies on (**A**) synthetic organic chemicals and eight controlled time series on (**B**) biological larvicides were included, conducted between years 1975 and 2004. Squares represent the relative effectiveness of individual studies, where square size represents the weight given to the study in the meta-analysis, with error bars representing 95% CIs; diamonds represent the pooled effects from random effects (RE) sub-group and meta-analyses.

Across six eligible studies, synthetic organic chemicals were effective in reducing anopheline larval numbers regardless of their application frequency: the pooled reduction was 77% in five CTS studies (95% CI 86.6, 61.4, p < 0.0001) and 72% in one CITS study (95% CI 89.5, 26.9, p = 0.01) (Fig. 2A, Table 2). Pyrethroids (e.g. deltamethrin) and organophosphates (e.g. temephos and iodenphos) provided a high level of control, reducing up to 90% larvae in Asian and African rice fields. Across the CTS studies, vector density evaluation usually occurred at least 6 times, from 24 h to 2 months after insecticide application. One quantitative study included adult malaria vectors as an outcome but found no association between iodenphos and human biting rate^26^ (Supplementary Table 3). However, qualitatively, two studies in the US observed significant reductions in adult density upon using organophosphates (Supplementary Table 4)^27,28^.

Across all eligible studies, biological larvicides were mostly applied once or twice throughout an experiment and vector density evaluation usually occurred at least three times, from 24 h to 6 weeks after insecticide application. Pooling across all frequencies and timings of applications, bacterial larvicides were associated with 60% fewer riceland anopheline larvae in eight CTSs (95% CI 71.8, 43.1, p < 0.0001, Fig. 2B) but not in two CITSs (Table 2). The most effective larvicides were *Bti-based*, against *An. gambiae* s.s. in Madagascar and *An. sinensis* in Taiwan. Three studies showed that bacterial larvicides produced greater reductions in the density of older immature stages, reducing pupae by up to 91%, followed by 67% in late and 47% in early-stage larvae (Supplementary Table 2). In studies evaluating the combination of bacterial larvicides and rice-fish systems compared to no intervention, the results were mixed: two CITSs showed an 88% reduction in anopheline immatures (95% CI 95.0, 71.3, p = 0.003), whilst two CTSs showed no association (Table 2). According to six studies that were only analysed qualitatively, both bacterial larvicide cum insect growth regulators and insect growth regulators alone could reduce riceland *An. quadrimaculatus* (Supplementary Table 4).

### Biological control

The simultaneous cultivation of rice and fish was effective in reducing the abundance of anopheline immatures, where a pooled reduction of 82% was found in three CTSs (95% CI 91.4, 60.2, p < 0.0001) and 87% in three CITSs (95% CI 93.9, 72.7, p = 0.001). In South Korea, *Aphycypris chinensis* (belonging to the carp or minnow family) was highly effective in reducing *An. sinensis* immatures whilst *Tilapia mossambicus* was not^29,30^. In Liberia, rice fields stocked with *T. nilotica* were associated with 88% lower *An. gambiae* s.l. numbers. *Gambusia affinis* (mosquitofish) were more effective against *An. freeborni* in the US when higher rates were stocked (Table 2). Other forms of biological control, including copepods, *Azolla* (mosquito fern) and neem, were not associated with lower numbers of anopheline larvae in rice fields (Table 2).

### Rice cultivation practices

**Figure 3:**
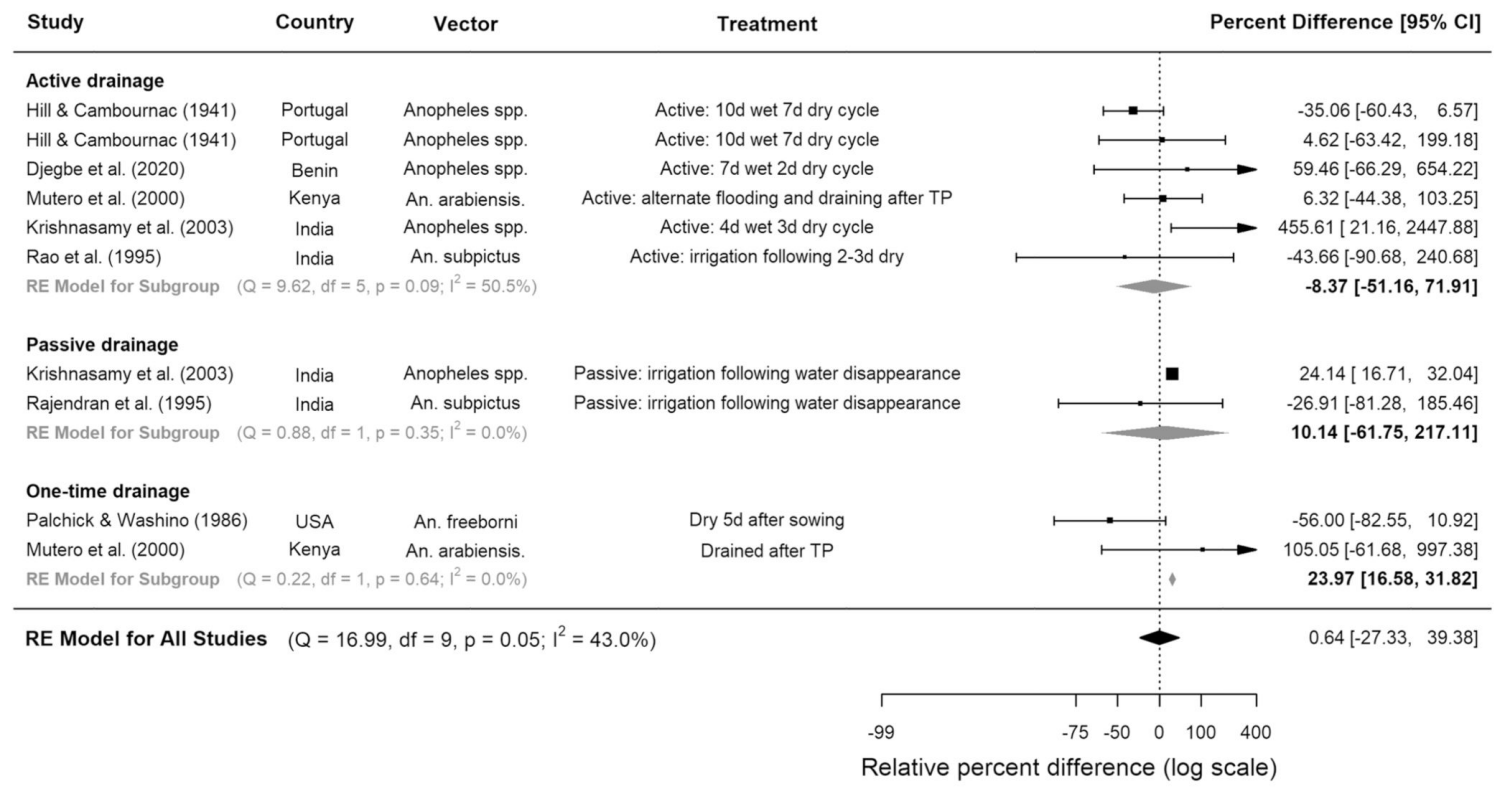
The effect of different intermittent irrigation techniques on larval densities of *Anopheles* vectors in rice fields. Seven studies were included, conducted between years 1936 and 2016. Squares represent the relative effectiveness of individual studies, where square size represents the weight given to the study in the metaanalysis, with error bars representing 95% CIs; diamonds represent the pooled effects from random effects (RE) sub-group and meta-analyses.

All trials experimenting with rice cultivation practices were CTS studies. Compared to continuously flooded fields, water management techniques involving drying intervals were not consistently associated with lower densities of anopheline immatures (Fig. 3, Table 3). When separated into subgroups according to type of drainage, neither active (where water is removed by drainage into canals) nor passive (where water is lost through evaporation or percolation) intermittent irrigation was associated with reduced larval densities, but one-time drainage was associated with 24% higher densities (95% CI 16.6, 31.8, p < 0.0001, 2 studies, Fig. 3). When immature abundance was separated into developmental stages, it was revealed that although intermittent irrigation was not associated with significant reductions in early instar larvae, it reduced the abundance of late instars by a pooled estimate of 35% in four CTS studies (95% CI 43.5, 24.0, p = 0.002, Supplementary Table 5). In one Kenyan study, draining during transplanting followed by active intermittent irrigation was associated with a 35% reduction in late stage larvae, but a 770% increase in early stage larvae^31^. In another study, based in China, qualitative analysis showed that intermittent irrigation provided good control of *An. sinensis* larvae^32^ (Supplementary Table 4).

Increasing water height in rice fields was associated with 96% higher *An. freeborni* larval densities in the US (95% CI 83.0–110.0, p < 0.0001, one study, Table 3). One study comparing water management systems found no association between efficient drainage systems and either anopheline larvae abundance or human biting rate^33^ (Supplementary Table 5).

Studies that examined the effect of rice cultivation practices other than water management methods were scarce (Table 3). One study in Japan observed that varying rice plant heights was not associated with larval numbers^34^. A study in India showed that plant density, regardless of rice variety, did not affect anopheline larval densities^35^. Palchick and Washino (1986) observed that using herbicides for weed control, compared to no weed control, was associated with 77% (95% CI 65.7, 89.9, p < 0.0001) higher larval numbers^36^. On the other hand, pesticides were associated with a 76% reduction (95% CI 88.8, 50.2, p = 0.001) of anopheline larvae in Indonesia^37^. Different processes in land preparation seemed to affect mosquito numbers: whilst levelling had no effect, rice plots that were minimally tilled were associated with a 65% reduction (95% CI 85.5, 14.1, p = 0.02, one study) compared to those with deep tillage^38^.

### Rice yield and water consumption

Agronomic outcomes were not measured in the eligible studies that investigated larviciding and biological control in rice fields; they were only measured in four studies assessing intermittent irrigation (Table 4). A meta-analysis of the four studies revealed that water management techniques alternative to continuous flooding did not significantly affect rice yield. In Portugal, however, Hill and Cambournac (1941) observed a 15% increase in yield (95% CI 0.5, 31.9, p = 0.005)^39^. This study, combined with Krishnasamy et al. (2003), demonstrated that intermittent irrigation (active or passive) reduced water use significantly, saving around 15% (95% CI 24.0, 5.7, p = 0.002)^40^.

### Scalability of technologies

Of 47 quantitative and qualitative studies, 13 studies (11 quantitative and 2 qualitative) included intervention readiness in their discussions (Supplementary Table 6). One study showed that using MSFs seemed to be appropriate for small-scale rice farmers, whilst larvicides were not economical, especially at an individual field basis^27,41,42^. Sundaraj and Reuben (1991) stated that in order to increase acceptance, labour-saving operations must be developed^42^. Fish, on the other hand, seemed to be well-accepted as an additional source of income and protein^43,44^. *Azolla* was also popular amongst rice farmers, not only because rice yields increased, but also because weed pressure halved^45^. Neem, however, needed to be more affordable and commercially available to promote large-scale use^45^.

Discussions on the scalability of intermittent irrigation were mixed: in Portugal and China, it was well-accepted and promoted by the government due to increased yield and decreased water consumption^32,39^. In India, farmers held different views: whilst convinced of intermittent irrigation based on water conservation, they doubted their own ability to organise water distribution and wanted the supervision of a government agency^46^. Moreover, its efficacy was dependent on farmer practices and a lot of effort was still required to change practices on a large scale^40^. In Kenya, intermittent irrigation could not be recommended to farmers as rice yield was not increased significantly, required more labour and had no apparent advantage on water consumption^31^.

## Discussion

We investigated whether ricefield mosquito larval control and/or rice cultivation practices are associated with malaria vector densities through a systematic review and meta-analysis. Forty-seven experimental studies were eligible for inclusion in the qualitative analysis and thirty-three studies were eligible for the meta-analysis. It was demonstrated that the use of fish, chemical and biological larvicides in rice fields were effective in controlling larval malaria vector densities at all developmental stages. Intermittent irrigation, however, could only significantly reduce late-stage larvae. Based on a limited number of studies, meta-analyses on other forms of larval control such as monomolecular surface films (MSFs), neem, copepods and *Azolla* failed to demonstrate any consistent reduction in anopheline numbers. Similarly, rice cultivation practices such as plant variety and density, type of levelling and pesticide application were not generally associated with reduced malaria vectors. Nonetheless, in one study, minimal tillage was observed to reduce average numbers of larvae throughout a cropping season. In another study, herbicide application increased larval abundance over a 4-week period, as did one-time drainage in a third study.

Despite their different modes of action, the use of chemical and bacterial larvicides and MSFs were all relatively effective measures of larval control in rice fields, varying between a 57% to 76% reduction in vector abundance compared to no larviciding. Their effects were highest (often reaching 100% reduction) only shortly following application but did not persist for longer than two weeks. These larvicides mostly had short residual half-lives because they were applied to paddy water which was naturally not completely stagnant: there was a small but constant process of water loss (through drainage, evapotranspiration and percolation) and replacement through irrigation. Hence, even with a residual formulation, weekly re-application would be needed for sustained control^47–50^. This would be very labour- and cost-intensive to scale-up, to ensure that larvicides are evenly distributed across vast areas (even at plot/sub-plot level) throughout at least one 5-month long ricegrowing season per year^42,51^. Aerial application (including unmanned aerial vehicles), although widely used in the US and Europe, is unlikely to be a feasible delivery system for smallholders in SSA, even in large irrigation schemes^26,27,48,49^. Furthermore, if synthetic organic chemicals were to be considered for riceland malaria vector control, their management in the current landscape of insecticide resistance across Africa must be considered.

Biological control using fish was found to be, in general, slightly more effective than (chemical, bacterial and MSF) larviciding. The degree of effectiveness was dependent on the fish species and their feeding preferences: surface-feeding, larvivorous species provided better anopheline control than bottom-feeding selective feeders^4,43^. Selecting the most suitable fish for local rice fields is not straightforward; many criteria need to be considered^4,52,53^. Generally, fish were well-received by rice farmers, perceived to contribute to increased yield by reducing weeds and pests and providing fertiliser through excrement^43,44^. This was reportedly also observed in Guangxi, China, where a certain proportion of the field had to be deepened into a side-trench where the fish could take shelter when the fields were drained. Even with this reduction in rice production area, carp rearing still increased yields by 10% and farmer’s income per hectare by 70%^53^. Unfortunately, none of the eligible studies in this review had included yield or water use as an outcome. Future entomological studies need to measure these critical agronomic variables so that studies of vector control in rice can be understood by, and transferred to, agronomists. In SSA, irrigated rice-fish farming can be scaled up provided that an inventory of fish species suitable for specific locations is available and that water is consistently available in fields (an important limiting factor in African irrigation schemes)^54^. Lessons can be learnt from successful large-scale rice-fish systems in Asia, where they have served as win–win solutions for sustainable food production and malaria control^16,55^.

Overall, there was only limited evidence that intermittent irrigation is effective at reducing late-instar anopheline larvae in rice fields. This finding contrasts with prior reviews, which found mixed results (regardless of larval stage) but emphasised that success was site-specific^4,17,56^. This contrast is presumably due to the inclusion criteria of our systematic review. These reviews excluded studies in various geographical settings and some older studies that reported successful anopheline control with intermittent irrigation but lacked either a contemporaneous control arm, adequate replication or adequate differentiation between culicines and anophelines^16,57–61^. It seems, from our review, that intermittent irrigation does not prevent the recruitment of early instars (and in one case, may have encouraged oviposition^31^) but tends to prevent their development into late-stage immatures. This important conclusion is, however, based only on four studies; more evidence is urgently needed where future trials should consider the basic principles of modern trials with adequate replication, controls and differentiation between larval instars and species.

Generally, it is observed that drainage, passive or active, did not reliably reduce overall numbers of mosquito immatures. In India and Kenya, closer inspection revealed that soils were not drying sufficiently, so any stranded larvae were not killed^31,46^. Highlighted by van der Hoek et al.^29^ and Keiser et al.^17^, water management in rice fields is very dependent on the physical characteristics of the soil and the climate and is most suited to places that not only favour rapid drying, but also have a good control of water supply^17,56^. Moreover, repeated drainage, although directed against mosquitoes, can also kill their aquatic predators^62^. Since mosquitoes can re-establish themselves in a newly flooded rice field more quickly than their predators, intermittent irrigation with more than a week between successive drying periods can permit repeated cycles of mosquito breeding without any predation pressure. Its efficacy against malaria vectors is therefore highly reliant on the timing of the wetting and drying periods. Further site-specific research on timing, especially with regards to predator–prey interactions within the rice agroecosystem, is required to find the perfect balance.

Another limitation in intermittent irrigation is that it cannot be applied during the first two to three weeks following transplanting, because rice plants must remain flooded to recover from transplanting shock. Unfortunately, this time coincides with peak vector breeding. Thus, other methods of larval control would be required to fill this gap. To agronomists, intermittent irrigation provides benefits to farmers, as it does not penalise yield but significantly reduces water consumption. Nonetheless, farmer compliance seems to be variable, especially in areas where water availability is inconsistent and intermittent irrigation would potentially require more labour^31,32,39^. Importantly, rice farmers doubted their ability to coordinate water distribution evenly amongst themselves, suggesting that there may be sharing issues, as in the “tragedy of the commons”^63^. Instead, they said that they preferred to have an agreed authority to regulate water^46^.

No general conclusions could be made on the effect on malaria vectors of other rice cultivation practices (apart from water management) because only one study was eligible for each practice. Nevertheless, these experiments on pesticide application, tillage and weed control, as well as another study on plant spacing (not eligible since glass rods were used to simulate rice plants), do illustrate that small changes in agronomic inputs and conditions can have considerable effects on mosquito densities, not just rice yield^36,38,64^. Moreover, in partially- or shallowly-flooded plots, the larvae are often concentrated in depressions (usually footprints), suggesting that rice operations which leave or remove footprints (e.g. hand-weeding, drum seeders, levelling) will influence vector breeding^4^.

Our study has some important limitations. First, in most trials, the units of intervention were replicate plots of rice, and success was measured as a reduction in larval densities within treated plots. This design focuses on the identification of effective and easy-to-implement ways of growing rice without growing mosquitoes, on the assumption that higher vector densities are harmful. However, from a public health perspective, the need for epidemiological outcomes is often, and reasonably, stressed^22,65^. Nonetheless, from a farmers’ perspective, it is also important to consider whether the vectors emerging from their rice fields significantly contribute to the local burden of malaria and to determine how this contribution can be minimised. There is evidence that riceland vectors do increase malaria transmission, since human biting rates are much higher in communities living next to rice schemes than their non-rice counterparts^66^ and that additional riceland vectors may intensify transmission and malaria prevalence in rice communities^15^. Hence, when investigating how rice-attributed malaria risk can be minimised, mosquito abundance as measured in the experimental rice trials is a useful indicator of potential impact on epidemiological outcomes.

Second, larval density was not always separated into larval developmental stages. This can be misleading because some interventions work by reducing larval survival (but not by preventing oviposition) and development to late instars and pupae. Therefore, an intervention could completely eliminate late-stage larvae and pupae but have little effect on the total number of immatures. This was illustrated in our meta-analyses of intermittent irrigation in Table 3 and Supplementary Table 5, and could have been the case for some studies that failed to demonstrate consistent reductions in overall anopheline numbers but did not differentiate between larval instars^34,45,67–69^. We infer that when monitoring mosquito immatures in rice trials, it is important to distinguish between larval instars and pupae. Pupae should always be counted separately since its abundance is the most direct indicator of adult productivity^70^.

Third, experimental trials rarely reported the timing of intervention application or accounted for different rice-growing phases, or “days after transplantation”, in the outcome. Both aspects are important to consider since an intervention may be suited to control larvae during certain growth phases but not others. This is illustrated by Djegbe et al.^38^, where, compared to deep tillage, minimal tillage could significantly reduce larvae during the early stages of rice cultivation but not during tillering and maturation^38^. In contrast, other interventions, such as *Azolla* and predatory copepods, took time to grow and accumulate, and were more effective during the later stages of a rice season^45,67,71^. This differentiation is important because it can identify components that could potentially form a complementary set of interventions against riceland malaria vectors, each component being effective at different parts of the season. Since rice fields, and hence the dynamics of riceland mosquito populations, vary from place to place, this set of interventions must also be robust. Special attention must be paid to the early stages of rice cultivation, particularly the first few weeks after transplanting (or sowing), since, with many vector species, a large proportion of adult mosquitoes are produced during this time.

Fourth, the analysis of entomological counts is often inadequate. Many studies failed to provide the standard deviation (or any other measure of error) for larval counts and could not be included in the quantitative analysis. Often, due to the extreme (and not unexpected) variability of larval numbers, sample sizes were insufficient to calculate statistically significant differences between treatments. Fifth, a high risk of bias was found across both CTS and CITS studies, including high heterogeneity and some publication bias. Study quality was, in general, a shortcoming and limited the number of eligible studies for certain interventions, including intermittent irrigation. Moreover, there are conspicuous a priori reasons for bias in such experimental trials: trial locations are frequently chosen to maximise the probability of success.

Finally, few studies were conducted in African countries, where the relationship between rice and malaria is most important because of the efficiency, and the “rice-philic” nature, of the vector *An. gambiae* s.l.^15^. In particular, there was a lack of studies on the effectiveness and scalability of biological control and rice cultivation practices. There is also very little information (particularly social science studies) on the views and perspectives of African rice farmers on mosquitoes in rice and interventions to control them^72,73^.

In the future, as malaria declines (particularly across SSA), the contribution of rice production to increased malaria transmission is likely to become more conspicuous^15^. Unless this problem is addressed, rice growing will probably become an obstacle to malaria elimination. Current default methods of rice production provide near-perfect conditions for the larvae of African malaria vectors. Therefore, we need to develop modified ricegrowing methods that are unfavourable to mosquitoes but still favourable for the rice. Although larviciding and biological control may be appropriate, their unsustainable costs remain the biggest barrier to uptake amongst smallholder farmers. Future investigations into riceland vector control should pay more attention to interventions that may be useful to farmers.

Supported by medical entomologists, agronomists should lead the research task of identifying cultivation methods that achieve high rice productivity whilst suppressing vector productivity. Rice fields are a major global source of greenhouse gases, and agronomists have responded by successfully developing novel cultivation methods that minimise these emissions while maintaining yield. We need the same kind of response from agronomists, to achieve malaria control co-benefits within rice cultivation. At present, only a few aspects of rice cultivation have been investigated for their effects on mosquitoes, and the potential of many other practices for reducing anopheline numbers are awaiting study. Due to the spatial and temporal heterogeneity of rice agroecosystems, it is likely that no single control method can reduce mosquito numbers throughout an entire cropping season and in all soil types and irrigation methods. Thus, effective overall control is likely to come from a combination of local, site-specific set of complementary methods, each of which is active and effective during a different phase of the rice-growing season.

## Conclusions

Our findings suggest that whilst larviciding, fish and intermittent irrigation can reduce the breeding of malaria vectors in rice fields, their effectivness is sensitive to environmental conditions and highly dependent on the timing and frequency of both intervention application and sampling. There is a lack of experimental studies on the interactions between these factors and their effects on anopheline larval densities, especially during different parts of a rice-growing season. Such studies are needed to find a robust combination of rice cultivation practices that do not exacerbate, and can potentially control, malaria vector production throughout an entire cropping season. To do this, the agricultural sector needs to take the lead, and take responsibility, for the deadly mosquitoes produced by agriculture. Therefore, long-term alliances between the agricultural and health sectors are required, not only to develop effective methods to control mosquitoes without compromising rice yields, but also to encourage intervention uptake and adoption by farmers through agricultural extension systems.

## Methods

A systematic review and a meta-analysis were conducted to assess how specific rice cultivation practices and mosquito control methods affect malaria vector abundance, rice yield and water consumption. Recommendations of the Preferred Reporting Items for Systematic reviews and Meta-Analyses (PRISMA) were followed. The study was not registered with the International Prospective Register of Systematic Reviews because it did not consider outcomes from human subjects and mosquitoes are not considered animal subjects. KC and JL did the systematic search, selected studies for inclusion and extracted relevant information. Any disagreements were resolved by LT. Data were extracted by KC and a 10% sub-sample was randomly selected for validation by JL.

### Eligibility criteria

This systematic review was concerned with mosquito populations. The intervention term encompassed a wide range of measures related to rice-growing practices (rice variety, plant density, land preparation method, crop establishment method and water management technique as well as application of fertilisers, herbicides and pesticides) and potential larval control (synthetic organic chemicals, oils and surface agents, biological larvicides, insect growth regulators, fish, nematode, *Azolla*, neem).

Studies were included if they measured effects on the relative density of larvae and pupae of malaria vectors (measured by area samplers, sweeping or standard dipping techniques) or the relative density of adult malaria vectors (measured by human landing catch, CDC light trap, pyrethrum spray catch, odour-baited traps or emergent traps). The secondary outcomes of interest were agronomic measures including rice yield (in tonnes per hectare) and water consumption (defined as the amount of used for rice cultivation in cubic metres).

Only experimental study designs were considered; (1) controlled time series trials (CTSs), with the unit of allocation being a rice plot and at least two replications per arm; (2) controlled interrupted time series studies (CITSs), with a contemporaneous control group and at least two replications per arm (Supplementary Fig. 1). Studies were included only if they reported data collected from experimental rice fields; laboratory and semifield studies were excluded. Studies were excluded if a control arm was absent and if the follow-up periods in each arm differed.

### Search strategy

PubMed, Embase, Global Health, SCOPUS, Web of Science, AGRIS, GreenFILE, TRIP database, BASE, ProQuest Dissertations & Theses Global, and EThoS were searched from 5 to 10th October 2020 to identify all relevant studies, using specified search terms (Supplementary Table 7). The search was restricted to published studies dated from 1900, and in English and French language. Proceedings from the following conferences were also searched: the MIM Pan-African Malaria Conferences, Pan-African Mosquito Control Association, American Society of Tropical Medicine and Hygiene, American Mosquito Control Association, Society for Vector Ecology and Agriculture for Nutrition and Health Academy Week. Reference lists of all relevant identified studies and published reviews were also searched. Authors and colleagues in the field were contacted for any additional references.

### Data extraction

From each eligible study, the following information were extracted into a pre-designed form: country, study setting, study design, intervention(s), control group, outcome(s), sampling, sample size, and vector(s). Any statements concerning the adoptability or scalability of the intervention by rice farmers were also extracted. If relevant data was unclear or not reported, study authors were contacted for clarification.

### Risk of bias

Risk of bias for CTSs and CITSs was assessed using the Effective Practice and Organisation Care (EPOC) tool^74^. If a sufficient number of studies were included in the meta-analysis, funnel plots were constructed and Egger’s test for funnel plot asymmetry were conducted to assess risk of publication bias^75^.

### Data analysis

Analyses were structured by (1) the type of intervention, (2) outcome and (3) study design. All eligible studies were included in a qualitative analysis. If sufficient data to calculate crude effects was reported (i.e. standard deviations or 95% confidence intervals), studies were also included in a quantitative analysis. Postintervention data were considered only up to the end of a rice-growing season, marked by harvest. Each outcome (entomological and agronomic) was combined in separate meta-analyses.

Analyses were conducted in R (version 4.1.2)^20^. For both entomological (count) and agronomic outcomes in CTSs, measures of effect (relative percent difference) were calculated by back-transforming the log-transformed ratio of means. For CITSs, relative percent differences were calculated by fitting a quasi-Poisson regression (due to overdispersion in larval counts) to pre- and post-intervention period (i.e. interruption) whilst using the control as an offset term to adjust for trend^21^. For CTSs, means were compared between study arms. Where there were multiple measurements over several time points, these were averaged. Grouped by study design, random effects models were then used to calculate pooled measures of effect and their 95% CI to illustrate the effect of each intervention on each outcome^19,13^. Heterogeneities were analysed using the *I^2^* statistic, and to reduce the extent of heterogeneity, random effects models were used.

## Supplementary Material

Additional File 1

## Figures and Tables

**Table 1:** Interventions tested by studies included in the qualitative and quantitative analysis (n = 47* studies), stratified by publication period and geographical region.

	Larviciding				Biological control		Environmental management/ rice cultivation practices		Total
	Oils and surface agents	Synthetic organic chemicals	Biological larvicides	Insect growth regulator	Fish	Copepod, *Azolla*, neem	Irrigation	Other: land preparation, water height, plant height	
**Publication period**									
1941–1950		1					2		3
1951–1960		1							1
1961–1970									0
1971–1980	1	3			1				5
1981–1990		3	*	1	4*		2*	2	21
1991–2000	1	1*	4*		2	3*	3*	2	16
2001–2010		1*	3*		1			2	7
2011–2021	1						1*	1*	3
**Geographical region**									
Africa	3	2*	3*		1*		1*	3*	13
South Asia		2	2*		1*	2*	4*	1	12
America		4*	9*	1	3	1	1*	2	21
East and SE Asia		2*	2*		3		1	1	9
Europe							1		1
Total	3	10	16	1	8	3	8	7	

* Studies with multiple comparisons that are treated separately here: Allen et al.^48^, Bolay and Trpis^44^, Djegbe et al.^38^, Kramer et al. (1988), Palchick and Washino^36^, Rajendran and Reuben^45^, Rao et al.^68^, Teng et al. (2005), and Yu et al. (1989).

**Table 2:** Summary of findings of meta-analyses of the effect of riceland mosquito control on *Anopheles* larval density (the number of larvae and pupae per dip or area sampler), arranged by the type of control, study design and geographical region. Significant values are in bold.

Study	Country	Predominant vector	Details of intervention (application method, rate, dose, frequency, timing, fish species)	Study design	Plot size (no. of replications*)	Relative percent difference (95% CI)
**Larviciding**						
**Surface agents**						
Reiter (1980)	Kenya	*An. gambiae* s.l	Monomolecular surface film (lecithin solution) at rate of 2.47 L/ha	CTS^1^	600 m^2^ (9)	–60.0 (–74.0, –38.5)
Reiter (1980)	Kenya	*An. gambiae* s.l	Monomolecular surface film (lecithin solution) at rate of 4.94 L/ha	CTS	600 m^2^ (15)	–57.1 (–76.3, –22.3)
Bukhari et al. (2011)	Kenya	*An. gambiae* s.l	Monomolecular surface film (Aquatain, silicone-based) at 1 ml/m^2^ (1st application) and at 2 ml/ m^2^ (2nd application)	CTS	2000 m^2^ (6)	–29.1 (–79.0, + 138.7)
				**RE model for all studies**		**– 57.2 (– 69.4, – 40.3)**
Karanja et al. (1994)	Kenya	*An. arabiensis*	Monomolecular surface film (Arosurf MSF) at 4 L/ ha every 14 days	CITS^2^	100 m^2^ (4)	–91.6 (–99.9,+ 486.3)
**Synthetic organic chemicals**						
Allen et al. (2008)	USA	*An. quadrimaculatus*	Lambda-cyhalothrin, aerial application at 5.5 g AI/ha, once (1 ×) prior permanent flooding	CTS	13–15 ha (2)	–9.3 (–40.9, + 39.0)
Ravoahangimalala et al. (1994)	Madagascar	*An. gambiae* s.s	Deltamethrin emulsionable concentrate 25 5 g/ ha, 1 ×	CTS	58–110m^2^ (2)	–92.7 (–95.4, –88.5)
Ravoahangimalala et al. (1994)	Madagascar	*An. gambiae* s.s	Deltamethrin emulsionable concentrate 25 12.5 g/ ha, 1 ×	CTS	43–58 m^2^ (2)	–92.9 (–96.5, –85.8)
Yap and Ho (1977)	Malaysia	*Anopheles* spp.	Chlorpyrifos (Dursban) at 14 gm/ha, 1 ×	CTS	(3)	–79.0 (–91.8, –46.5)
Yap and Ho (1977)	Malaysia	*Anopheles* spp.	Chlorpyrifos (Dursban) at 28 gm/ha, 1 ×	CTS	(3)	–75.2 (–90.6, –34.5)
Yap and Ho 1977)	Malaysia	*Anopheles* spp.	Chlorpyrifos (Dursban) at 56 gm/ha, 1 ×	CTS	(3)	–67.8 (–82.3, –41.4)
Yap and Ho (1977)	Malaysia	*Anopheles* spp.	Organophosphorus (Dowco-214) at 56 gm/ ha, 1 ×	CTS	(3)	–68.0 (–83.6, –37.5)
Yap et al. (1982)	Malaysia	*Anopheles* spp.	Temephos (Abate 500E) 60 gm/ha, 1 ×	CTS	69–365 m^2^ (2)	–56.3 (–86.8, + 45.0)
Yap et al. (1982)	Malaysia	*Anopheles* spp.	Temephos (Abate 500E) 100 gm/ha, 1 ×	CTS	69–365 m^2^ (2)	–77.0 (–93.0, –24.4)
Yap et al. (1982)	Malaysia	*Anopheles* spp.	Temephos (Abate 500E) 200 gm/ha, 1 ×	CTS	69–365 m^2^ (2)	–61.2 (–89.5, + 43.2)
Teng et al. (2005)	Taiwan	*An. sinensis*	Temephos (Abate 1-SG) at 1 ppm, 2 × (20 day interval)	CTS	119–194 m^2^ (4)	–91.2 (–97.5, –69.3)
				**RE model for all studies**		**– 73.1 (– 83.8, – 55.4)**
Kamel et al. (1972)	^**E**^gyp^**t**^	*An. pharoensis*	Iodofenphos (NUVANOL N20U), aerial application at 1.5 L/ha, 1 ×	CITS	50–120 ha (2)	–93.2 (–98.1, –76.2)
Kamel et al. (1972)	Egypt	*An. pharoensis*	Iodofenphos (NUVANOL N20U), aerial application at 3 L/ha, 1 ×	CITS	50–120 ha (2)	–50.2 (–83.3, + 49.0)
				**RE model for all studies**		**– 72.3 (– 89.5, – 26.9)**
**Biological larvicides**						
Allen et al. (2008)	USA	*An. quadrimaculatus*	*Bacillus thuringiensis* var. *israelensis* (*Bti*), AQUA-BACxt, aerial application at 108 L/ha on a 61-m swath, 1 ×	CTS	13–15 ha (3)	–60.8 (–86.9, + 17.1)
Dennett et al. (2001)	USA	*An. quadrimaculatus*	*Bacillus sphaericus* (*Bs*), VectoLex WDG, aerial application at 1.68 kg/ ha, 1 ×	CTS	2000 m^2^ (2)	–8.6 (–24.1,+ 10.1)
Dennett et al. (2001)	USA	*An. quadrimaculatus*	*Bs*, VectoLex WDG, aerial application at 0.56 kg/ ha, 1 ×	CTS	2000 m^2^ (2)	–11.1 (–24.2, + 4.2)
Ravoahangimalala et al. (1994)	Madagascar	*An. gambiae* s.s	*Bti*, Teknar HP-D liquid concentrate, at 0.6 L/ ha, 1 ×	CTS	58–68 m^2^ (2)	–81.1 (–86.1, –74.4)
Ravoahangimalala et al. (1994)	Madagascar	*An. gambiae* s.s	*Bti*, Teknar HP-D liquid concentrate, at 1.25 L/ ha, 1 ×	CTS	58–78 m^2^ (2)	–87.7 (–92.7, –79.5)
Ravoahangimalala et al. (1994)	Madagascar	*An. gambiae* s.s	*Bti*, Teknar HP-D liquid concentrate, at 12.5 L/ ha, 1 ×	CTS	58–87 m^2^ (2)	–93.2 (–96.1, –88.0)
Sundaraj and Reuben (1991)	India	*An. subpictus*	*Bs*, Biocide-S 1593 M, at 2.2 kg/ha, 1 × after transplantation	CTS	440 m^2^ (3)	–74.9 (–90.5, –33.5)
Sundaraj and Reuben (1991)	India	*An. subpictus*	*Bs*, Biocide-S 1593 M, at 4.3 kg/ha, 1 × after transplantation	CTS	440 m^2^ (3)	–75.8 (–92.4, –22.7)
Kramer et al. (1988)	USA	*An. freeborni*	*Bti*, Vectobac (200 ITU/ mg), at 6 kg/ha, 2 × (when mosquito densities were high)	CTS	1000 m^2^ (3)	–56.1 (–81.3, + 2.8)
Teng et al. (2005)	Taiwan	*An. sinensis*	*Bti*, Vectobac G, at 1 g/m^2^, 2 × (20 day interval)	CTS	119–194 m^2^ (4)	–83.8 (–94.9, –48.6)
Teng et al. (2005)	Taiwan	*An. sinensis*	*Lagenidium giganteum* T, 1.5 ppm and 30 oz/acre, 2 × (20 day interval)	CTS	119–194 m^2^ (4)	–38.5 (–80.7, + 95.7)
Teng et al. (2005)	Taiwan	*An. sinensis*	*Lagenidium giganteum* A, 1.5 ppm and 30 oz/acre, 2 × (20 day interval)	CTS	119–194m^2^ (4)	+ 1.3 (–69.0, + 231.3)
Balaraman et al. 1983)	India	*An. subpictus*	*Bti* serotype H-14 (VCRC B-17), with dose 27 × 10^5^ spores/mL, 3 ×	CTS	1000 m^2^ (5)	–75.8 (–87.0, –55.0)
McLaughlin et al. (1982)	USA	*An. crucians*	*Bti*, H-14 (Abbott 6108b 300 T.U./mg), at 6.0 kg/ ha, 3 ×	CTS	30 m^2^ (3)	–42.3 (–58.1, –20.4)
McLaughlin et al. (1982)	USA	*An. crucians*	*Bti*, H-14 (Abbott 6108b 300 T.U./mg), at 3.0 kg/ ha, 3 ×	CTS	30 m^2^ (3)	–60.8 (–66.2, –54.5)
McLaughlin et al. (1982)	USA	*An. crucians*	*Bti*, H-14 (Abbott 6108b 300 T.U./mg), at 1.5 kg/ ha, 3 ×	CTS	30 m^2^ (3)	–42.3 (–58.5, –19.7)
McLaughlin et al. (1982)	USA	*An. crucians*	*Bti*, H-14 (Biochem-Bactimos 666 1800 T.U./mg), at 1.0 kg/ha, 3 ×	CTS	30 m^2^ (3)	–30.0 (–48.8, –4.3)
McLaughlin et al. (1982)	USA	*An. crucians*	*Bti*, H-14 (Biochem-Bactimos 666 1800 T.U./mg), at 0.5 kg/ha, 3 ×	CTS	30 m^2^ (3)	–29.1 (–41.4, –14.2)
McLaughlin et al. (1982)	USA	*An. crucians*	*Bti*, H-14 (Biochem-Bactimos 666 1800 T.U./mg), at 0.25 kg/ha, 3 ×	CTS	30 m^2^ (3)	–23.2 (–38.4, –4.4)
				**RE model for all studies**		**– 60.0 (– 71.8, – 43.1)**
Bolay & Trpis (1989)	Liberia	*An. gambiae* s.l	*Bti*, Teknar HP-D, at 0.1 g/m^2^	CITS	150 m^2^ (3)	–75.8 (–96.0, + 46.3)
Yu et al. (1993)	S. Korea	*An. sinensis*	*Bti*, H-14 (Bactis-P), at 0.1 kg/ha	CITS	1000 m^2^ (6)	–67.6 (–97.0, + 251.1)
				**RE model for all studies**		**– 76.3 (– 95.4, + 21.9)**
**Larviciding and biological control**						
**Bacterial larvicide and fish**						
Kramer et al. (1988)	USA	*An. freeborni*	*Bti*, Vectobac (200 ITU/ mg), at 6 kg/ha +*Gambusia affinis* at 1.1 kg/ha	CTS	1000 m^2^ (3)	–31.0 (–68.3, + 50.3)
Kramer et al. (1988)	USA	*An. freeborni*	*Bti*, Vectobac (200 ITU/ mg), at 6 kg/ha +*G. affinis* at 3.4 kg/ha	CTS	1000 m^2^ (3)	–82.8 (–91.9, –63.4)
				**RE model for all studies**		**– 65.7 (– 91.2, + 34.2)**
Bolay & Trpis (1989)	Liberia	*An. gambiae* s.l	*Bti*, Teknar HP-D, at 0.1 g/ m^2^+*Tilpania nilotica* (300)	CITS	150 m^2^ (3)	–88.1 (–96.1, –63.9)
Yu & Lee (1989)	S. Korea	*An. sinensis*	*Bti*, H-14, at 1 kg/ ha +*Aplocheilus latipes* at 2/m^2^	CITS	150 m^2^ (2)	–67.0 (–79.8, –46.2)
				**RE model for all studies**		**– 88.0 (– 95.0, – 71.3)**
**Biological control**						
**Fish**						
Kramer et al. (1988)	USA	*An. freeborni*	*G. affinis* at 1.1 kg/ha	CTS	1000 m^2^ (3)	–77.7 (–88.2, –56.1)
Kramer et al. (1988)	USA	*An. freeborni*	*G. affinis* at 3.4 kg/ha	CTS	1000 m^2^ (3)	–88.6 (–94.2, –77.9)
Victor et al. (1994)	India	*An. subpictus*	3 indigenous carps (*Catla catla, labeo rohita, cirrhinus mrigala)* + 3 exotic carps *(Cyprinus carpio, Hypopthalmithys molitri, Ctenopharyngodon idella)*stocked at rate of 10,000/ha	CTS	400 m^2^ (3)	–51.6 (–76.2, –1.6)
Yu et al. (1981)	S. Korea	*An. sinensis*	*Aphycypris chinensis* (presence)	CTS	2000 m^2^ (2)	–92.2 (–97.3, –77.2)
				**RE model for all studies**		**– 81.5 (– 91.4, – 60.2)**
Bolay & Trpis (1989)	Liberia	*An. gambiae* s.l	*Tilapia nilotica* (n = 300)	CITS	150 m^2^ (3)	–87.8 (–96.0, –62.4)
Kim et al. (2002)	S. Korea	*An. sinensis*	*Tilapia mossambicus* at 2 fish/10 m^2^	CITS	300–600 m^2^(2–4)	–41.8 (–57.1, –20.9)
Kim et al. (2002)	S. Korea	*An. sinensis*	*A. chinensis* at 2 fish/10m^2^	CITS	300–600 m^2^(2–4)	–62.4 (–76.0, –41.2)
Kim et al. (2002)	S. Korea	*An. sinensis*	*T. mossambicus* at 2 fish/10m^2^+*A. chinensis* at 1/m^2^	CITS	300–600 m^2^ (2–4)	–55.1 (–72.6, –26.3)
Yu & Lee (1989)	S. Korea	*An. sinensis*	*A. latipes* at 2 fish/m^2^+*T. mossambicus* at 2/m^2^	CITS	150 m^2^ (2)	–73.4 (–80.5, –63.6)
				**RE model for all studies**		**– 87.1 (– 93.9, – 72.7)**
**Copepod**						
Marten et al. (2000)	USA	*An. Quadrimaculatus*	*Mesocyclops ruttneri* (n=500)	CTS	100 m^2^ (2)	–40.5 (–82.8, + 105.6)
***Azolla***						
Rajendran & Reuben (1991)	India	*An. subpictus*	*Azolla microphylla* introduced at rate 100 g/m^2^ on 5th DAT^3^	CTS	40 m^2^ (2)	–48.7 (–96.8, + 720.4)
Rajendran & Reuben (1991)	India	*An. subpictus*	*Azolla microphylla* introduced at rate 200 g/m^2^ on 5th DAT	CTS	40 m^2^ (2)	+ 45.6 (–89.0, + 1826.3)
				**RE model for all studies**		**-10.3 (-86.4, + 493.3)**
**Neem**						
Rao et al. (1995)	India	*An. subpictus*	Neem (Nimin) at 0.063 kg ai/ha	CTS	400 m^2^ (3)	–29.4 (–84.3, + 217.8)
Rao et al. (1995)	India	*An. subpictus*	Neem (Nimin)-coated urea at 0.063 kg ai/ ha + 62.5 kg urea/ha	CTS	400 m^2^ (3)	–34.0 (–74.4, + 70.4)
Rao et al. (1995)	India	*An. subpictus*	Neem-coated urea (Neemrich-1 80EC^4^) at 0.09 kg ai/ha	CTS	400 m^2^ (3)	–25.1 (–75.4, + 127.7)
Rao et al. (1995)	India	*An. subpictus*	As above + 62.5 kg urea/ha	CTS	400 m^2^ (3)	–33.2 (–83.5, + 171.2)
Rao et al. (1995)	India	*An. subpictus*	Neem-coated urea (Neemrich-1 80EC) at 0.12 kg ai/ha	CTS	400 m^2^ (3)	–27.0 (–81.5, + 187.4)
Rao et al. (1995)	India	*An. subpictus*	As above + 62.5 kg urea/ha	CTS	400 m^2^ (3)	–32.6 (–76.6, + 93.9)
				**RE model for all studies**		**– 30.7 (– 57.2, + 12.3)**
***Azolla* and neem**						
Rajendran & Reuben (1991)	India	*An. subpictus*	*Azolla microphylla* at 100 g/m^2^ on 5th DAT + neem cake powder 50 g/m^2^ on day of transplantation (TP)	CTS	40 m^2^ (2)	–53.9 (–96.5, + 528.2)
**Neem and water management technique**						
Rao et al. (1995)	India	*An. subpictus*	Neem (Nimin)-coated urea at 0.063 kg ai/ ha + 62.5 kg urea/ ha + water allowed to stand 2.5–3.5 cm in the week following TP + from the second week, plots were dried for 2–3 days before re-irrigation	CTS	400 m^2^ (3)	–27.5 (–90.1,+ 430.6)
Rao et al. (1995)	India	*An. subpictus*	Neem-coated urea (Neemrich-1 80EC) at 0.09 kg ai + 62.5 kg urea/ ha + water allowed to stand 2.5–3.5 cm in the week following TP + from the second week, plots were dried for 2–3 days before re-irrigation	CTS	400 m^2^ (3)	–43.7 (–93.3, + 370.7)
				**RE model for all studies**		**– 35.6 (– 84.9, + 175.2)**

* The number of plots per treatment group. ^1^CTS: Controlled time series. ^2^CITS: Controlled interrupted time series. ^3^DAT: Days after transplanting. ^4^EC: Emulsifiable concentrate.

**Table 3:** Summary of findings of meta-analyses of the effect of rice cultivation practices on *Anopheles* larval density (the number of larvae and pupae per dip or area sampler), arranged by the type of control, study design and geographical region. Significant values are in bold.

Study	Country	Predominant vector	Comparison	Plot size (no. of replications)	Relative percent difference (95% CI)
**Intermittent irrigation**					
Palchick and Washino (1986)	USA	*An. freeborni*	Drained 5 DAS^1^, water depth raised to 3–5 inches until 60 DAS, then to 6–8 inches for rest of season	2800–3800 m^2^ (3)	+ 24.1 (+ 16.7, + 32.0)
Hill and Cambournac (1941)	Portugal	*Anopheles*	10 day wet, 7 day dry cycle*	100 m^2^ (4)	–35.1 (–60.4, + 6.6)
Hill and Cambournac 1941	Portugal	*Anopheles*	10 day wet, 7 day dry cycle*	2000 m^2^ (4)	+ 4.6 (–63.4, + 199.2)
Djegbe et al. (2020)	Benin	*Anopheles*	7 day wet, 2 day dry cycle*	16.5 m^2^ (3)	–56.0 (–82.5, + 10.9)
Mutero et al. (2000)	Kenya	*An. arabiensis*	Flooded before TP, drained during TP^2^, flooded after TP	750 m^2^ (4)	+ 6.3 (–44.4, + 103.3)
Mutero et al. (2000)	Kenya	*An. arabiensis*	Flooded before TP, drained during TP, alternately flooded and drained after TP	750 m^2^ (4)	+ 455.6 (+ 21.2, + 2448.0)
Krishnasamy et al. (2003)	India	*An. subpictus*	4d wet, 3d dry cycle* (rotational water supply)	Varying sizes (5)	+ 59.6 (–66.3, + 654.2)
Krishnasamy et al. (2003)	India	*An. subpictus*	Irrigation to 5 cm one day after disappearance of ponded water in fields	Varying sizes (5)	+ 105.1 (–61.7, + 997.4)
Rajendran et al. (1995)	India	*An. subpictus*	2.5 cm depth maintained for the first 10–14 DAT^3^. Fields subsequently dried out and re-irrigated to 5 cm depth immediately after all standing water had disappeared (3-5d after irrigation stopped)	16.2–22.3 ha (2)	–26.9 (–81.3, + 185.5)
Rao et al. (1995)	India	*An. subpictus*	Water allowed to stand 2.5–3.5 cm in the week following TP + from the second week, plots were dried for 2–3 days before re-irrigation	400 m^2^ (3)	–43.7 (–90.7, + 240.7)
			**RE model for all studies**		**+ 0.6 (– 27.3, + 39.4)**
**Control of water depth**					
Palchick and Washino (1986)	USA	*An. freeborni*	Medium: water level 3–5 inches during first 60d then raised to 6–8 inches vs shallow: water level 1–2 inches during first 60d then to 6–8 inches	2800–3800 m^2^ (3)	+ 89.7 (+ 77.7, + 102.4)
Palchick and Washino (1986)	USA	*An. freeborni*	Deep: 6–8 inches all season vs shallow: water level 1–2 inches during first 60d then to 6–8 inches	2800–3800 m^2^ (3)	+ 103.4 (+ 89.1, + 118.9)
					**+ 96.0 (+ 83.0, + 110.0)**
**Water management system**					
Sogoba et al. (2007)	Mali	*An. gambiae* s.l	*Hors-casier* plot sector (no technical assistance in irrigation system and therefore lack efficient drainage systems) vs. *casier* plot sector (renovated irrigation systems)	1000 m^2^ (4)	+ 113.4 (–50.9, + 827.1)
**Rice variety**					
Takagi et al. (1996)	Japan	*An. sinensis*	Tall rice (98.5 cm) vs short rice (45 cm)	1500 m^2^ (2)	+ 150.0 (–66.1, + 1745.1)
**Rice variety and plant spacing**					
Victor and Reuben (2000)	India	*An. subpictus* & *An. vagus*	ADT36 (short duration variety of 110 days) at 60 hills/m^2^(20 × 15 cm) vs. 80 hills/m^2^(15 × 10 cm)	40 m^2^ (4)	–49.1 (–94.8, + 396.5)
Victor and Reuben (2000)	India	*An. subpictus* & *An. vagus*	IR50 (short duration variety of 110 days) at 60 hills/ m^2^ (20 × 15 cm) vs. 80 hills/m^2^(15 × 10 cm)	40 m^2^ (4)	–77.9 (–97.0, + 60.8)
Victor and Reuben (2000)	India	*An. subpictus* & *An. vagus*	IR20 (medium duration variety of 120 days) at 60 hills/m^2^(20 × 15 cm) vs. 80 hills/m^2^ (15 × 10 cm)	40 m^2^ (4)	–62.0 (–95.2, + 202.5)
			**RE model for all studies**		**– 66.3 (– 90.0, + 13.4)**
**Weed control**					
Palchick and Washino (1986)	USA	*An. freeborni*	Weed controlled by herbiciding vs. no weed control	2800–3800 m^2^(3)	+ 77.4 (+ 65.7, + 89.9)
**Agricultural insecticide**					
Martono (1988)	Indonesia	*An. aconitus*	Organophosphorous compound (Basudin 60 EC) used to control paddy pests (such as *Trvporvza* spp., *Leptocorsica acuta* and *Nilaparvata Lugens)* at 960 ppm	250 m^2^ (2)	–76.4 (–88.8, –50.2)
**Land preparation**					
Djegbe et al. (2020)	Benin	*Anopheles* spp.	Minimal tillage (tillage depth < 15 cm) vs. deep tillage	16.5 m^2^ (3)	–64.7 (–85.5, –14.1)
Djegbe et al. (2020)	Benin	*Anopheles* spp.	Normal levelling vs. abnormal levelling	16.5 m^2^ (3)	–12.8 (–65.2, + 118.5)

* Water is applied to the field so that it is wet for X days and left for X days to dry before being irrigated again. ^1^DAS: Days after seeding. ^2^ TP: Transplanting.^3^ DAT: Days after transplanting. ^4^ EC: Emulsifiable concentrate.

**Table 4:** Summary of findings of meta-analyses of the association between different types of rice cultivation practices and agronomic outcomes. Significant values are in bold.

Study	Country	Comparison	Plot size (no. of replications)	Outcome	Relative percent difference (95% CI)
**Water management techniques**					
Hill and Cambournac (1941)	Portugal	10 day wet, 7 day dry cycle*	2000 m^2^ (4)	Rice yield	+ 15.1 (+ 0.5, + 31.9)
Mutero et al. (2000)	Kenya	Flooded before TP^1^, drained during TP, flooded after TP	750 m^2^ (4)	Rice yield	– 7.9 (– 18.0, + 3.3)
Mutero et al. (2000)	Kenya	Flooded before TP, drained during TP, alternately flooded and drained after TP	750 m^2^ (4)	Rice yield	– 9.5 (– 21.3, + 4.0)
Krishnasamy et al. (2003)	India	4 day wet, 3 day dry cycle* (rotational water supply)	Varying sizes (5)	Rice yield	+ 3.9 (– 0.7, + 8.7)
Krishnasamy et al. (2003)	India	Irrigation to 5 cm one day after disappearance of ponded water in fields	Varying sizes (5)	Rice yield	– 0.2 (– 5.5, + 5,4)
Rajendran et al. (1995)	India	2.5 cm depth maintained for the first 10–14 DAT^2^. Fields subsequently dried out and re-irrigated to 5 cm depth after all standing water had disappeared (3–5 day after irrigation stopped)	162,000–223,000 m^2^ (2)	Rice yield	+ 2.4 (– 8.1, + 14.1)
			**RE model for all studies**		**+ 0.8 (**– **3.8, + 5.7)**
Hill and Cambournac (1941)	Portugal	10 day wet, 7 day dry cycle*	2000 m^2^ (4)	Water use	– 18.5 (– 30.0, – 5.1)
Krishnasamy et al. (2003)	India	4 day wet, 3 day dry cycle* (rotational water supply)	Varying sizes (5)	Water use	– 7.5 (– 10.5, - –4.5)
Krishnasamy et al. (2003)	India	Irrigation to 5 cm 1 day after disappearance of ponded water in fields	Varying sizes (5)	Water use	– 21.0 (– 23.8, – 18.0)
			**RE model for all studies**		– **15.4** (– **24.0**, – **5.7)**

* Water is applied to the field so that it is wet for X days and left for X days to dry before being irrigated again. ^1^TP: Transplanting. ^2^DAT: Days after transplanting.

## Data Availability

All the studies used in this study are published in the literature.
