# Supplementary material for: The control of malaria vectors in rice fields: a systematic review and meta-analysis
Source: Sci Rep. Author manuscript; Available in PMC 2022 Dec 1. (PMC9668837; doi:10.1038/s41598-022-24055-2)
Supplement: Additional File 1 [file EMS157181-supplement-Additional_File_1.pdf]

The control of malaria vectors in rice fields: A systematic review and meta-analysis

Supplementary Figure 1. Illustration of experimental study designs considered.

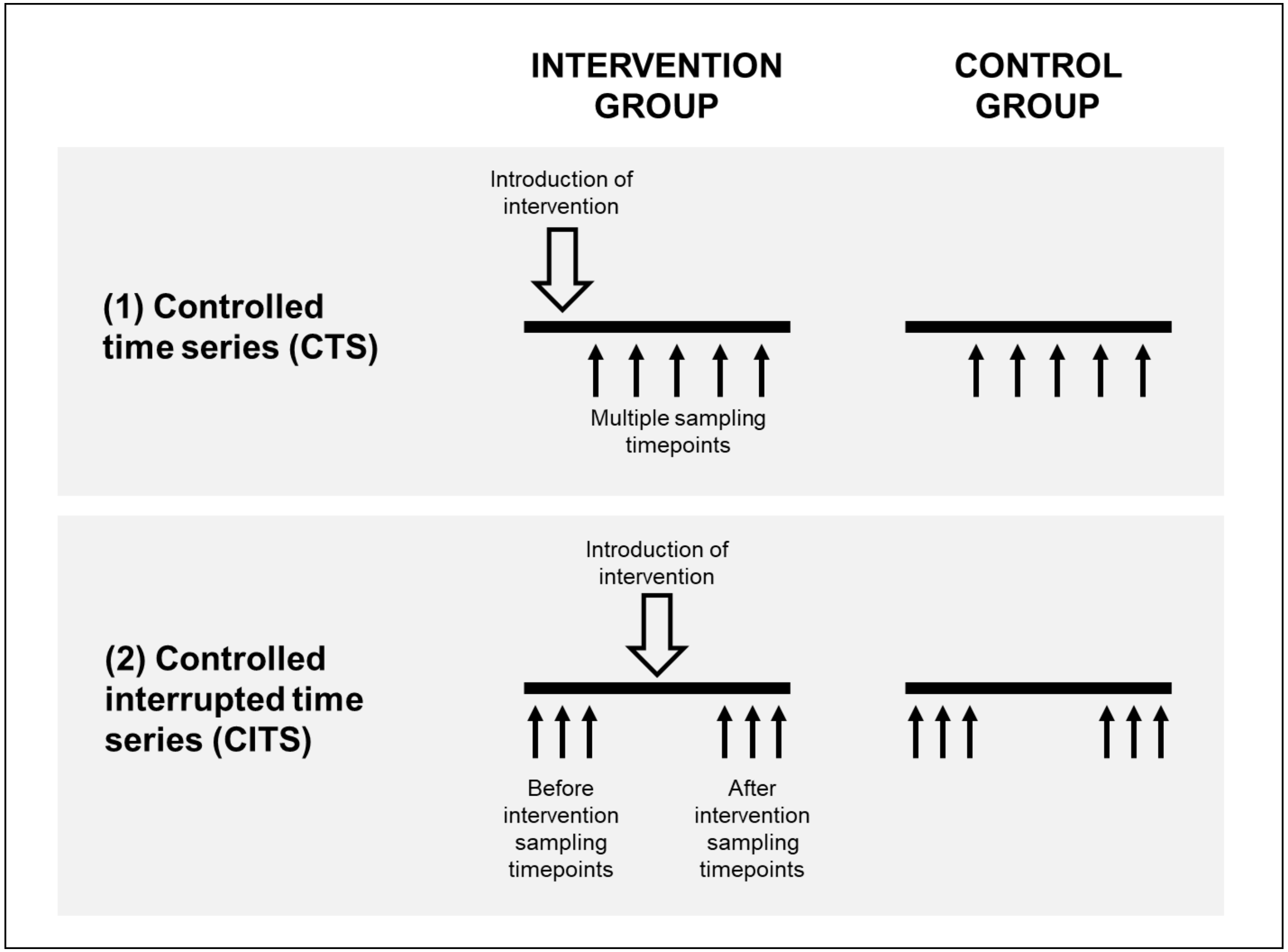

Supplementary Table 1. Risk of bias assessment for studies included in the quantitative analysis (controlled time series and controlled before-after studies, n=33).

Reference		Allocation sequence generation	Allocation concealment	Baseline outcome measurements	Baseline features	Incomplete outcome data	Blinding (performance)	Blinding (detection)	Contamination	Selective outcome reporting	Overall assessment (Low / some concerns / high)
	Low	Intervention randomly allocated	Allocation conducted by investigators on all units at the start of the study	Outcomes were measured prior to the intervention and no important differences were present across study groups, or if imbalanced but appropriate adjusted analysis was performed	Features of the study and control areas are reported and similar	Missing outcome measures are unlikely to bias the results	Performance bias unlikely due to lack of knowledge of the allocated interventions	Outcomes were assessed blindly or outcomes are objective	It is unlikely that the control group received the intervention	No evidence that outcomes were selectively reported (all pre-specified outcomes are reported)	
	High	When a non-random method is used, and for non-randomised trials and controlled before-after studies	Technicians and investigators could foresee assignment, and for controlled before-after studies	Important differences were present and not adjusted for analysis	No report of characteristics in text or tables or if there are differences between control and intervention areas	Missing outcome data likely to bias the results	Performance bias likely due to knowledge of the allocated interventions	Outcomes were not assessed blindly	It is likely that the control group received the intervention	Some important outcomes are subsequently omitted from the results	
	Unclear	Not specified in the paper	Not specified in the paper	If randomised trials have no baseline measure of outcome	Not specified in the paper	Not specified in the paper (do not assume 100% follow-up unless stated explicitly)	Not specified in the paper	Not specified in the paper	-	Not specified in the paper	
Allen et al. 2008		Unclear	High	Low	Low	Low	High	High	Low	Low	Some concerns
Balaraman et al. 1983		Unclear	High	Unclear	Unclear	Low	High	High	Low	Low	High
Bolay & Trpis 1989		High	High	Low	Low	Low	High	High	Low	Low	Some concerns
Bukhari et al. 2011		High	High	Low	Low	Low	High	High	Low	Low	Some concerns
Dennett et al. 2001		Unclear	High	Low	Unclear	Low	High	High	Low	Low	High
Djegbe et al. 2020		Unclear	High	Unclear	Unclear	Low	High	High	Low	Low	High
Hill & Cambournac 1941		Unclear	High	Low	Low	Low	High	High	High	High	High
Kamel et al. 1972		High	High	High	High	Low	High	High	High	Low	High
Karanja et al. 1994		High	High	Low	Low	Low	High	High	Low	Low	Some concerns
Kim et al. 2002		High	High	Low	Low	Low	High	High	Low	Low	Some concerns
Kramer et al. 1988		Low	High	Low	High	Low	High	Low	Low	Low	Some concerns
Krishnasamy et al. 2003		Unclear	High	High	Unclear	Low	High	High	Low	High	High
Marten et al. 2000		Unclear	High	Low	Low	Low	High	High	Low	Low	Some concerns
Martono 1988		High	High	Low	Low	Low	High	High	Low	Low	Some concerns
McLaughlin et al. 1982		Unclear	High	Low	Low	Low	High	High	Low	Low	Some concerns
Mutero et al. 2000		Low	High	Low	Low	Low	High	High	Low	Low	Some concerns

Reference		Allocation sequence generation	Allocation concealment	Baseline outcome measurements	Baseline features	Incomplete outcome data	Blinding (performance)	Blinding (detection)	Contamination	Selective outcome reporting	**Overall assessment** (Low / some concerns / high)
	Low	Intervention randomly allocated	Allocation conducted by investigators on all units at the start of the study	Outcomes were measured prior to the intervention and no important differences were present across study groups, or if imbalanced but appropriate adjusted analysis was performed	Features of the study and control areas are reported and similar	Missing outcome measures are unlikely to bias the results	Performance bias unlikely due to lack of knowledge of the allocated interventions	Outcomes were assessed blindly or outcomes are objective	It is unlikely that the control group received the intervention	No evidence that outcomes were selectively reported (all pre-specified outcomes are reported)	
	High	When a non-random method is used, and for non-randomised trials and controlled before-after studies	Technicians and investigators could foresee assignment, and for controlled before-after studies	Important differences were present and not adjusted for analysis	No report of characteristics in text or tables or if there are differences between control and intervention areas	Missing outcome data likely to bias the results	Performance bias likely due to knowledge of the allocated interventions	Outcomes were not assessed blindly	It is likely that the control group received the intervention	Some important outcomes are subsequently omitted from the results	
	Unclear	Not specified in the paper	Not specified in the paper	If randomised trials have no baseline measure of outcome	Not specified in the paper	Not specified in the paper (do not assume 100% follow-up unless stated explicitly)	Not specified in the paper	Not specified in the paper	-	Not specified in the paper	
Palchick & Washino 1986		Unclear	High	Unclear	Unclear	Low	High	High	Low	Low	High
Rajendran & Reuben 1991		Unclear	Unclear	Low	Low	Low	High	High	High	Low	High
Rajendran et al. 1995		Unclear	High	Low	Low	Low	High	High	Low	Low	Some concerns
Rao et al. 1995		Low	High	Low	Low	Low	High	High	Low	Low	Some concerns
Ravoahangimalala et al. 1994		Unclear	Unclear	Low	Low	Low	High	High	Low	Low	Some concerns
Reiter 1980		Unclear	High	Low	Low	Low	High	High	Low	Low	Some concerns
Sogoba et al. 2007		High	High	High	Unclear	Low	High	High	High	Low	High
Sundaraj & Reuben 1991		Unclear	High	Low	Low	Low	High	High	Low	Low	Some concerns
Takagi et al. 1996		Unclear	High	Low	Unclear	Low	High	High	Low	Low	Some concerns
Teng et al. 2005		Low	High	Low	Low	Low	High	High	Low	Low	Some concerns
Victor et al. 1994		Unclear	High	Low	Low	Low	High	High	Low	Low	Some concerns
Victor & Reuben 2000		Low	High	Unclear	Low	Low	High	High	Low	Low	Some concerns
Yap et al. 1982		Unclear	Unclear	High	Low	Low	High	High	Low	Low	Some concerns
Yu et al. 1981		Unclear	Unclear	Unclear	High	Low	High	High	High	Low	High
Yu & Lee 1989		High	High	Low	Low	Low	Low	High	Low	Low	Some concerns
Yu et al. 1993		High	High	Low	Low	Low	High	High	Low	Low	Some concerns

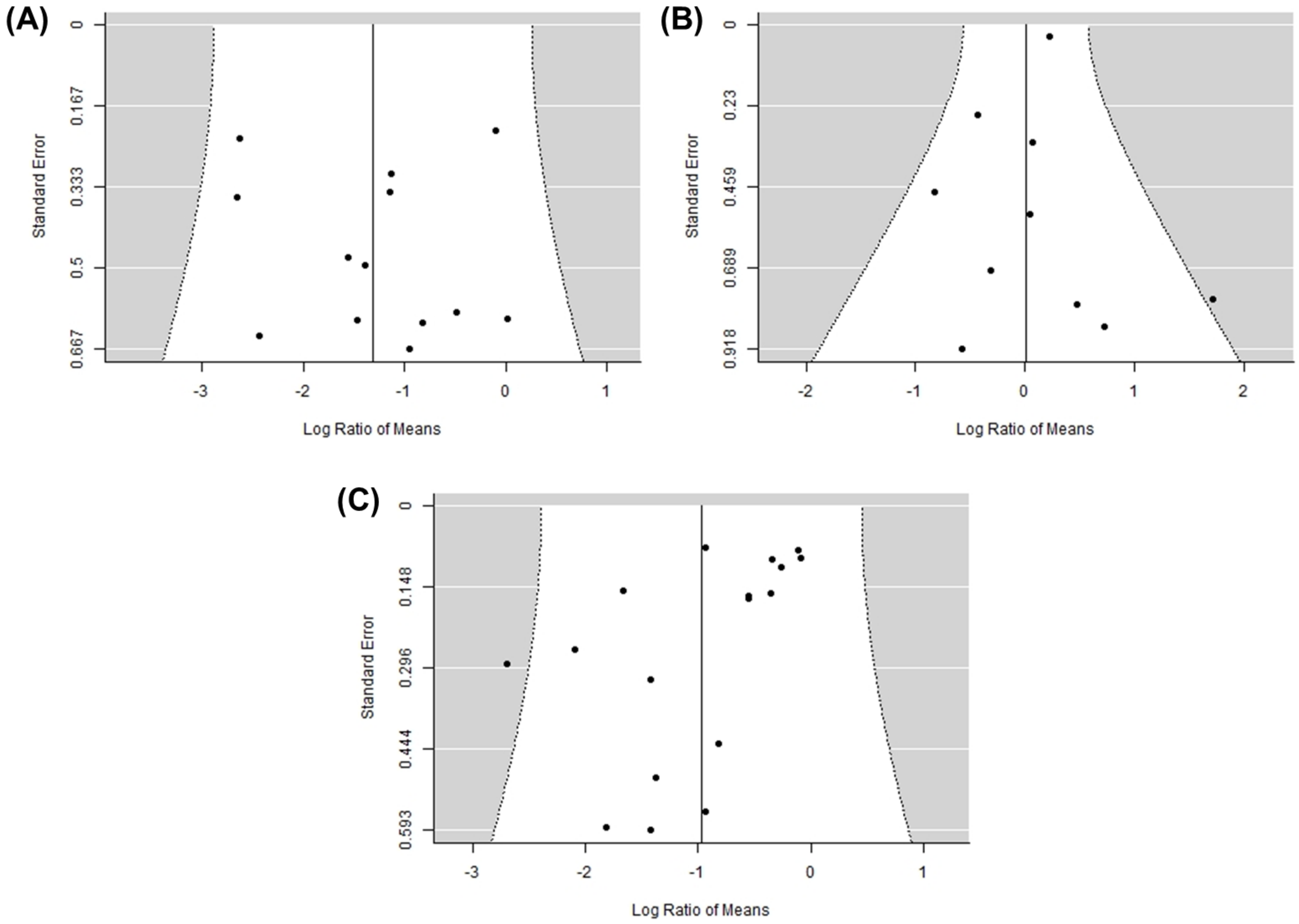

Supplementary Figure 2. Funnel plots assessing publication bias in the meta-analyses of the effect of riceland mosquito control. Specifically, **(A)** synthetic organic chemicals (test for funnel plot asymmetry: z = 0.54, p = 0.59), **(B)** water management techniques (z = 0.44, p = 0.66) and **(C)** bacterial larvicides (z = -2.26, p = 0.024), on *Anopheles* larval density.

Supplementary Table 2. Summary of findings of meta-analyses of the effect of riceland mosquito control on *Anopheles* larval density, arranged by the type of control, larval stage, study design and geographical region.

Study	Country	Vector	Details of intervention (application method, rate, dose, fish species)	Study design	Plot size (no. of replications)	Larval stage	Percent difference (95% CI)
**Larviciding**							
Oils and surface agents							
Bukhari et al. 2011	Kenya	*An. gambiae* s.l.	Monomolecular surface film (Aquatain, silicone-based) at 1 ml/m^2 (1st app.) and at 2 ml/m^2 (2nd app.)	CTS	2000 m^2 (6)	Early	-24.3 (-89.0, +422.8)
Reiter 1980	Kenya	*An. gambiae* s.l.	Monomolecular surface film (lecithin solution) at rate of 2.47 L/ha	CTS	600 m^2 (9)	Early	-52.1 (-74.5, -9.9)
							**-49.9 (-72.5, -8.8)**
Karanja et al. 1994	Kenya	*An. arabiensis*	Monomolecular surface film (Arosurf MSF) at 4 L/ha every 14 days	CITS	100 m^2 (4)	Early	**-93.5 (-99.8, 114.2)**
Bukhari et al. 2011	Kenya	*An. gambiae* s.l.	Monomolecular surface film (Aquatain, silicone-based) at 1 ml/m^2 (1st app.) and at 2 ml/m^2 (2nd app.)	CTS	2000 m^2 (6)	Late	-32.5 (-71.6, +60.6)
Reiter 1980	Kenya	*An. gambiae* s.l.	Monomolecular surface film (lecithin solution) at rate of 2.47 L/ha	CTS	600 m^2 (9)	Late	-59.5 (-73.0, -39.2)
							**-54.9 (-70.0, -32.4)**
Bacterial larvicide							
Balaraman et al. 1983	India	*An. subpictus*	*Bti* serotype H-14 (VCRC B-17), with dose 27 x 10^5 spores/ml	CTS	1000 m^2 (5)	Early	-75.8 (-88.8, -47.9)
Dennett et al. 2001	USA	*An. quadrimaculatus*	*Bacillus sphaericus* (*Bs*), VectoLex WDG, aerial application at 1.68 kg/ha	CTS	2000 m^2 (2)	Early	-7.3 (-31.7, +25.8)
Dennett et al. 2001	USA	*An. quadrimaculatus*	*Bs,* VectoLex WDG, aerial application at 0.56 kg/ha	CTS	2000 m^2 (2)	Early	-17.3 (-35.9, +6.8)
Sundaraj & Reuben 1991	India	*An. subpictus*	*Bs,* Biocide-S 1593M, at 2.2 kg/ha	CTS	440 m^2 (3)	Early	-65.5 (-87.3, -6.0)
Sundaraj & Reuben 1991	India	*An. subpictus*	*Bs,* Biocide-S 1593M, at 4.3 kg/ha	CTS	440 m^2 (3)	Early	-65.2 (-89.7, +17.3)
							**-46.7 (-67.4, -6.3)**
Balaraman et al. 1983	India	*An. subpictus*	*Bti* serotype H-14 (VCRC B-17), with dose 27 x 10^5 spores/ml	CTS	1000 m^2 (5)	Late	-66.5 (-88.3, -3.7)
Dennett et al. 2001	USA	*An. quadrimaculatus*	*Bs,* VectoLex WDG, aerial application at 1.68 kg/ha	CTS	2000 m^2 (2)	Late	-14.5 (-26.4, -0.8)
Dennett et al. 2001	USA	*An. quadrimaculatus*	*Bs,* VectoLex WDG, aerial application at 0.56 kg/ha	CTS	2000 m^2 (2)	Late	-11.0 (-25.5, +6.3)
Sundaraj & Reuben 1991	India	*An. subpictus*	*Bs,* Biocide-S 1593M, at 2.2 kg/ha	CTS	440 m^2 (3)	Late	-89.1 (-96.9, -62.0)
Sundaraj & Reuben 1991	India	*An. subpictus*	*Bs,* Biocide-S 1593M, at 4.3 kg/ha	CTS	440 m^2 (3)	Late	-92.3 (-97.3, -77.9)
							**-67.2 (-88.0, -10.7)**
Balaraman 1983	India	*An. subpictus*	*Bti* serotype H-14 (VCRC B-17), with dose 27 x 10^5 spores/ml	CTS	1000 m^2 (5)	Pupae	-95.7 (-99.1, -78.2)
Dennett et al. 2001	USA	*An. quadrimaculatus*	*Bs*, VectoLex WDG, aerial application at 1.68 kg/ha	CTS	2000 m^2 (2)	Pupae	-3.1 (-6.3, +0.3)
Dennett et al. 2001	USA	*An. quadrimaculatus*	*Bs,* VectoLex WDG, aerial application at 0.56 kg/ha	CTS	2000 m^2 (2)	Pupae	-1.7 (-5.0, +1.7)
Sundaraj & Reuben 1991	India	*An. subpictus*	*Bs,* Biocide-S 1593M, at 2.2 kg/ha	CTS	440 m^2 (3)	Pupae	-99.0 (-99.8, -93.9)
Sundaraj & Reuben 1991	India	*An. subpictus*	*Bs,* Biocide-S 1593M, at 4.3 kg/ha	CTS	440 m^2 (3)	Pupae	-99.4 (-99.9, -94.8)
							**-91.1 (-99.0, -22.0)**

Supplementary Table 3. Summary of findings of meta-analyses of the effect of riceland mosquito control and rice-growing techniques on *Anopheles* human biting rate (HBR), arranged by the type of control or intervention.

Study	Country	Predominant vector	Details of intervention (application method, rate, dose, fish species)	Study design	Plot size (no. of replications)	Outcome	Relative percent difference (95% CI)
**Larviciding**							
Synthetic organic chemicals							
Kamel et al. 1972	Egypt	*An. pharoensis*	Iodofenphos (NUVANOL N20U), aerial application at 1.5-3 L/ha	CITS	500,000 m^2 - 1,200,000 m^2	HBR indoors (HLC)<br>HBR outdoors (HLC)	-73.4 (-94.1, +5.4)<br>-52.0 (-80.2, +15.9)
Water management system							
Sogoba et al. 2007	Mali	*An. gambiae* s.l.	*Hors-casier* plot sector (no technical assistance in irrigation system and therefore lack efficient drainage systems) vs. *casier* plot sector (renovated irrigation systems)	CTS	1000 m^2 (4)	HBR	+44.2 (-50.7, +321.9)

Supplementary Table 4. The association between riceland mosquito control and larval density in studies included in the qualitative analysis (n=13).

Study	Country	Vector	Details of intervention (application method, rate, dose, fish species)	Study design	Plot size (no. of replications)	Outcome	Relative percent difference (%)
Synthetic organic chemicals							
Gahan & Nob 1955	USA	*An. quadrimaculatus*	Organophosphate (Parathion)	CTS	4000 m^2	Larval density	+57.9
Gahan & Nob 1955	USA	*An. quadrimaculatus*	Organophosphate (Bayer L 13/59)	CTS	4000 m^2	Larval density	+80.0
Gahan & Nob 1955	USA	*An. quadrimaculatus*	Organophosphate (Shell OS 2046)	CTS	4000 m^2	Larval density	+85.0
Magy 1949	USA	*An. freeborni*	Organochlorine (DDT), varying concentrations, aerial application	CITS	7284 ha	Larval density	-55.0 - -100.0
Washino et al. 1972	USA	*An. freeborni*	Organophosphate (Chlorpyrifos, Dursban)	CITS	1855 ha	Adult density	-11.1
Weathersbee et al. 1986	USA	*An. quadrimaculatus*	Mixture of malathion, heavy aromatic naphtha and resmethrin/PBO at 221.8 ml/min, aerial application and *Bti* ground application	CITS	1758 ha	Adult density	-59.9 12 hr post,<br>-6.8 36 hr post
Bacterial larvicides							
Bassi et al. 1989	USA	*An. quadrimaculatus*	*Bti* (Vectobac) at 78 ml AI/ha	CITS	61,000 m^2	Larval density	-72.3
Lacey & Inman 1985	USA	*An. crucians*	*Bti* (Vectobac) at 5.6 kg/ha, aerial application	CITS	4000 m^2	Larval density	-91.9
Lacey & Inman 1985	USA	*An. crucians*	*Bti* (Vectobac) at 11.2 kg/ha, aerial application	CITS	4000 m^2	Larval density	-94.2
Lacey & Inman 1985	USA	*An. crucians*	*Bti* (Vectobac) at 22.5 kg/ha, aerial application	CITS	4000 m^2	Larval density	-96.0
Lacey & Inman 1985	USA	*An. crucians*	*Bti* (Bactimos) at 2.8 kg/ha, hand application	CITS	4000 m^2	Larval density	-100.0
Lacey & Inman 1985	USA	*An. crucians*	*Bti* (Bactimos) at 5.6 5 kg/ha, aerial application	CITS	4000 m^2	Larval density	-100.0
Lacey & Inman 1985	USA	*An. crucians*	*Bti* (Bactimos) at 11.2 5 kg/ha, aerial application	CITS	4000 m^2	Larval density	-100.0
Lacey & Inman 1985	USA	*An. crucians*	*Bti* (Teknar) at 1.7 kg/ha, hand application	CITS	4000 m^2	Larval density	-100.0
Lacey & Inman 1985	USA	*An. crucians*	*Bti* (Teknar) at 3.0 kg/ha, aerial application	CITS	4000 m^2	Larval density	-100.0
Lacey & Inman 1985	USA	*An. crucians*	*Bti* (Teknar) at 7.5 kg/ha, aerial application	CITS	4000 m^2	Larval density	-100.0
Lacey & Inman 1985	USA	*An. quadrimaculatus* & *An. crucians*	*Bs* (isolate 2362), granular formulation	CTS	400 m^2 (3)	Larval density	-68.0

Study	Country	Vector	Details of intervention (application method, rate, dose, fish species)	Study design	Plot size (no. of replications)	Outcome	Relative percent difference (%)
Romi et al. 1993	Madagascar	*An. arabiensis*	*Bti* (Vectobac 12AS) at 1 kg/ha	CITS	180 m^2	Larval density	-89.0
Romi et al. 1993	Madagascar	*An. arabiensis*	*Bti* (Vectobac 12AS) at 0.6 kg/ha	CITS	260 m^2	Larval density	-100.0
Romi et al. 1993	Madagascar	*An. arabiensis*	*Bti* (Vectobac GR) at 10 kg/ha	CITS	220 m^2	Larval density	-100.0
Romi et al. 1993	Madagascar	*An. arabiensis*	*Bti* (Vectobac GR) at 5 kg/ha	CITS	410 m^2	Larval density	-100.0
Romi et al. 1993	Madagascar	*An. arabiensis*	*Bti* (ABG 6185) at 10 kg/ha	CITS	160 m^2	Larval density	-84.0
Romi et al. 1993	Madagascar	*An. arabiensis*	*Bti* (ABG 6185) at 5 kg/ha	CITS	160 m^2	Larval density	-57.0
Sandoski et al. 1985	USA	*An. quadrimaculatus*	*Bti* (Teknar) at 0.54 l/ha	CITS	17,000 m^2	Larval density	-97.9
Sandoski et al. 1985	USA	*An. quadrimaculatus*	*Bti* (Teknar) at 0.27 l/ha	CITS	17,000 m^2	Larval density	-94.4
Sandoski et al. 1985	USA	*An. quadrimaculatus*	*Bti* (Teknar) at 0.11 l/ha	CITS	17,000 m^2	Larval density	-93.0
Sandoski et al. 1985	USA	*An. quadrimaculatus*	*Bti* (Teknar) at 0.07 l/ha	CITS	17,000 m^2	Larval density	-71.1
Sandoski et al. 1985	USA	*An. quadrimaculatus*	*Bti* (Teknar) at 0.04 l/ha	CITS	17,000 m^2	Larval density	-21.8
Stark et al. 1983	USA	*An. quadrimaculatus*	*Bti* (serotype H-14 ABG-6108)	CTS	36 m^2	Larval density	-97.0
Insect growth regulator							
Kottkamp & Meisch 1985	USA	*An. quadrimaculatus*	Insect growth regulator (Bay Sir 8514) at 49 g ai/ha	CTS	36 m^2	Larval density	-73.7
Insect growth regulator and bacterial larvicide							
Bassi et al. 1989	USA	*An. quadrimaculatus*	Methoprene (insect growth regulator) and *Bti* (Duplex) at 78 ml AI/ha	CITS	61,000 m^2	Larval density	-95.1
Biological control							
Blaustein 1992	USA	*An. freeborni*	*G. affinis*: 10 gravid female, 10 male adults	CTS	83.6 m^2 (6)	Larval density	-7.3
Blaustein 1992	USA	*An. freeborni*	*L. cyanellus*: 20 adults	CTS	83.6 m^2 (6)	Larval density	+28.2
Blaustein 1992	USA	*An. freeborni*	*G. affinis*: 10 gravid female, 10 male adults and *L. cyanellus*: 20 adults	CTS	83.6 m^2 (6)	Larval density	-92.2
Hoy et al. 1971	USA	*An. freeborni*	*G. affinis* at stocking rate of 50 per acre	CTS	(3)	Larval density	-83.0
Hoy et al. 1971	USA	*An. freeborni*	*G. affinis* at stocking rate of 100 per acre	CTS	(3)	Larval density	-48.5
Hoy et al. 1971	USA	*An. freeborni*	*G. affinis* at stocking rate of 150 per acre	CTS	(3)	Larval density	-59.2
Hoy et al. 1971	USA	*An. freeborni*	*G. affinis* at stocking rate of 200 per acre	CTS	(3)	Larval density	-87.0
Hoy et al. 1971	USA	*An. freeborni*	*G. affinis* at stocking rate of 250 per acre	CTS	(3)	Larval density	-82.4
Hoy et al. 1971	USA	*An. freeborni*	*G. affinis* at stocking rate of 300 per acre	CTS	(3)	Larval density	-59.2
Intermittent irrigation							
Luh 1984	China	*An. sinensis*	Wet irrigation (a type of intermittent irrigation where fields are filled with a shallow layer of water that may disappear between 24-48 hours) vs. conventional irrigation	CTS		Larval density	-84.0 - -86.0

Supplementary Table 5. Summary of findings of meta-analyses of the effect of rice-growing techniques on *Anopheles* larval density, arranged by larval stage, study design and geographical region.

Study	Country	Vector	Comparison	Plot size (no. of replications)	Larval stage	Relative percent difference (95% CI)
Intermittent irrigation						
Hill & Cambournac 1941	Portugal	*Anopheles*	10d wet, 7d dry cycle	2000 m^2 (4)	Early	-36.9 (-64.7, +12.8)
Mutero et al. 2000	Kenya	*An. arabiensis*	Flooded before transplanting, drained during transplanting, flooded after transplanting	750 m^2 (4)	Early	+32.6 (+11.9, +57.2)
Mutero et al. 2000	Kenya	*An. arabiensis*	Flooded before transplanting, drained during transplanting, alternately flooded and drained after transplanting	750 m^2 (4)	Early	+770.6 (+113.4, +3450.9)
						**+69.9 (-57.3, +575.3)**
Hill & Cambournac 1941	Portugal	*Anopheles*	10d wet, 7d dry cycle	2000 m^2 (4)	Late	-32.6 (-64.5, +28.1)
Mutero et al. 2000	Kenya	*An. arabiensis*	Flooded before transplanting, drained during transplanting, flooded after transplanting	750 m^2 (4)	Late	-34.7 (-45.4, -21.7)
Mutero et al. 2000	Kenya	*An. arabiensis*	Flooded before transplanting, drained during transplanting, alternately flooded and drained after transplanting	750 m^2 (4)	Late	-35.0 (-51.9, -12.1)
Rajendran et al. 1995	India	*An. subpictus*	2.5 cm depth maintained for the first 10-14 DAT. Fields subsequently dried out and re-irrigated to 5 cm depth immediately after all standing water had disappeared (3-5d after irrigation stopped)	16.2 ha - 22.3 ha	Late	-28.8 (-71.0, +74.9)
						**-34.5 (-43.5, -24.0)**

Supplementary Table 6. The adoptability of each intervention according to rice farmers.

	Reference	Intervention or rice-growing method	Description
Interventions and rice-growing techniques that were accepted amongst local farmers	Karanja et al. 1994	Monomolecular surface film	Appropriate for small scale rice cultivation with no more than 1.5 hectares of paddy fields
	Victor & Reuben 1994	Fish	Net profit 2.5 times larger for rice cum fish than rice alone – can provide more income to farmers if successfully promoted
	Bolay & Trpis 1989	Fish	An additional source of protein in tropical countries with malnutrition
	Rajendran & Reuben 1991	Azolla	Grain yields not only increased by 9-14% in treated fields, but weed production halved, reducing need and cost of labour for weeding. Azolla also kept field moist when fields dried out. Farmers were thoroughly impressed.
	Hill & Cambournac 1941	Intermittent irrigation	Economic advantages outweigh disadvantages: increased cost in rebuilding field, relaying irrigation and drainage ditches, increased care necessary in preparation of fields, careful supervision of interruptions to irrigation but once fields are set up for practice, economic advantages resulting from increased yield and better conditions for labour stimulate interest on growers and practice spreading slowly
	Luh 1984	Intermittent irrigation	Rice yield increased by 13% and water consumption considerably lower. Method was therefore well-accepted and used on more than 100,000 ha in 1980
Interventions and rice-growing techniques that needed improvement for increased acceptability	Washino et al. 1972	Synthetic organic chemical	This cannot be done economically on an individual field basis. Low volume technique and synchronisation of large areas required to increase success
	Sundaraj & Reuben 1991	Bacterial larvicide	Application costs were too much; took one person 2.5 hours to spray 0.5 hectares. A less labour-intensive system must be developed (perhaps through multiple point source introduction into irrigation water)
	Rao et al. 1995	Neem	Strong incentive to adopt strategy for economic reasons – but commercial availability at an affordable price will promote large scale use
	Rajendran et al. 1995	Intermittent irrigation	Farmers were convinced of utility based on water conservation but doubted ability to organise equitable distribution during years of water scarcity, preferring neutral supervision of a government agency
	Mutero et al. 2000	Intermittent irrigation	Required more labour, which was already scarce since irrigated rice in the area was labour intensive. Provision of labour only expected if there was a real direct benefit in relation to rice yield, which there was not. Water saving would have benefited farmers during times of water scarcity but no apparent advantage in terms of water saving. The method could not be recommended for use by farmers unless rice yield increased significantly.
	Krishnasamy et al. 2003	Intermittent irrigation	Efficacy heavily dependent on farmer practices and considerable effort would be needed to change practices on a large scale
	Djegbe et al. 2020	Intermittent irrigation and land preparation	Need to assess on a wider scale the feasibility of implementing intermittent flooding with respect to farmer acceptance and required changes in irrigation system design and management

Supplementary Table 7. Search strategy in multiple databases (last search date = 10 October 2020).

Search set	Medline	EMBASE	Global Health	Web of Science
1	Exp malaria/	Exp malaria/	Exp malaria/	TS = malari*
2	Exp Anopheles/	Exp malaria control/	Exp anopheles/	TS = anophel*
3	Exp Culicidae/	Exp Anopheles/	Exp Culicidae/	TS = disease vector$
4	Exp mosquito control/	Exp mosquito control/	Exp disease vectors/	TS = mosquito*
5	Exp disease vectors/	Exp insect vector/	Exp entomology/	TS = mosquito control
6	Exp entomology/	Exp mosquito/	Exp medical entomology/	TS = malaria control
7	Malaria.tw.	Exp mosquito vector/	Malaria.ab.	1 OR 2 OR 3 OR 4 OR 5 OR 6
8	Malari*.tw.	Malaria.tw.	Malari*.ab.	TS = rice
9	Anophel*.tw.	Malari*.tw.	Anophel*.ab.	TS = “rice field$”
10	Mosquito*.tw.	Anophel*.tw.	Mosquito*.ab.	TS = “ricefield$”
11	Entomolog*.tw.	Mosquito*.tw.	Entomolg*.ab.	TS = “rice cultivat*”
12	Vector control.tw.	Entomolog*.tw.	1 or 2 or 3 or 4 or 5 or 6	TS = “rice grow*”
13	1 or 2 or 3 or 4 or 5 or 6	1 or 2 or 3 or 4 or 5 or 6 or 7	7 or 8 or 9 or 10 or 11	TS = “rice padd*”
14	7 or 8 or 9 or 10 or 11 or 12	8 or 9 or 10 or 11 or 12	12 or 13	TS = “rice irrigat*”
15	13 or 14	13 or 14	Exp rice/	TS = “water management”
16	Exp oryza/	Exp rice/	Exp flooded rice/	TS = weed*
17	Exp agriculture/	Exp agriculture/	Exp rice fields/	TS = fertili*
18	Herbicides/	Exp “irrigation (agriculture)”	Exp agriculture/	TS = “plant variet*”
19	Rice.tw.	Exp tillage/	Exp irrigation/	8 OR 9 OR 10 OR 11 OR 12 OR 13 OR 14 OR 15 OR 16 OR 17 OR 18
20	Rice field$.tw.	Rice.tw.	Rice.ab.	19 AND 7
21	Ricefield$.tw.	Rice field$.tw.	Rice field$.ab.	
22	Rice adj4 cultivat*.tw.	Ricefield$.tw.	Ricefield$	
23	Rice adj4 grow*.tw.	Rice adj4 cultivat*.tw.	Rice adj4 cultivat*.ab.	
24	Rice adj4 practice$.tw.	Rice adj4 grow*.tw.	Rice adj4 grow*.tw.	
25	Rice adj4 technique$.tw.	Rice adj4 practice$.tw.	Rice adj4 practice$.ab.	
26	Rice adj2 padd*.tw.	Rice adj4 technique$.tw.	Rice adj4 technique$.ab.	
27	Rice adj2 irrigat*.tw.	Rice adj2 padd*.tw.	Rice adj2 padd*.ab.	
28	Rice adj4 method*.tw.	Rice adj2 irrigat*.tw.	Rice adj2 irrigat*.ab.	
29	Monolayer.tw.	Rice adj4 method*.tw.	Rice adj4 method*.ab.	
30	Fertili*.tw.	Monolayer.tw.	Monolayer.ab.	
31	Weed*.tw.	Fertili*.tw.	Fertili*.ab.	
32	Plant adj3 variet*.tw.	Weed*.tw.	Weed*.ab.	
33	Water adj3 management.tw.	Plant adj3 variet*.tw.	Plant adj3 variet*.ab.	
34	16 or 17 or 18	Water adj3 management.tw.	Water adj3 management.ab.	
35	19 or 20 or 21 or 22 or 23 or 24 or 25 or 26 or 27 or 28 or 29 or 30 or 31 or 32 or 33	16 or 17 or 18 or 19	15 or 16 or 17 or 18 or 19	
36	34 or 35	20 or 21 or 22 or 23 or 24 or 25 or 26 or 27 or 28 or 29 or 30 or 31 or 32 or 33 or 34	20 or 21 or 22 or 23 or 24 or 25 or 26 or 27 or 28 or 29 or 30 or 31 or 32 or 33 or 34	
37	15 and 36	35 or 36	35 or 36	
38		15 and 37	14 and 37	
